# Accuracy of parameter estimation for auto-regulatory transcriptional feedback loops from noisy data

**DOI:** 10.1098/rsif.2018.0967

**Published:** 2019-04-03

**Authors:** Zhixing Cao, Ramon Grima

**Affiliations:** School of Biological Sciences, the University of Edinburgh, Max Born Crescent, Edinburgh EH9 3BF, UK

**Keywords:** inference, chemical master equation, gene regulatory networks

## Abstract

Bayesian and non-Bayesian moment-based inference methods are commonly used to estimate the parameters defining stochastic models of gene regulatory networks from noisy single cell or population snapshot data. However, a systematic investigation of the accuracy of the predictions of these methods remains missing. Here, we present the results of such a study using synthetic noisy data of a negative auto-regulatory transcriptional feedback loop, one of the most common building blocks of complex gene regulatory networks. We study the error in parameter estimation as a function of (i) number of cells in each sample; (ii) the number of time points; (iii) the highest-order moment of protein fluctuations used for inference; (iv) the moment-closure method used for likelihood approximation. We find that for sample sizes typical of flow cytometry experiments, parameter estimation by maximizing the likelihood is as accurate as using Bayesian methods but with a much reduced computational time. We also show that the choice of moment-closure method is the crucial factor determining the maximum achievable accuracy of moment-based inference methods. Common likelihood approximation methods based on the linear noise approximation or the zero cumulants closure perform poorly for feedback loops with large protein–DNA binding rates or large protein bursts; this is exacerbated for highly heterogeneous cell populations. By contrast, approximating the likelihood using the linear-mapping approximation or conditional derivative matching leads to highly accurate parameter estimates for a wide range of conditions.

## 1. Introduction

In recent years, it has been shown that a significant percentage of genes in bacteria and yeast are auto-regulated [1–3], i.e. a transcription factor activates or represses the expression of its own gene. We here choose to focus on negative auto-regulation (repression) because this motif confers significant advantages to cellular function including the reduction of intrinsic noise [4] and the speeding up of the response time [5]. It is also the case that the molecular mechanism of circadian oscillators relies on negative autoregulation of gene expression [6,7]. Given the widespread availability of experimental data on the number of mRNAs and proteins at the single cell level [8–11], a natural question is how can we use these data to infer the rate constants and other relevant parameters of negative auto-regulatory transcriptional feedback loops.

A number of early studies used rate equations to identify the underlying network structure of gene regulatory networks or to infer rate constants [12,13]. However, clearly this is not the ideal framework since rate equations are deterministic while it is well known that gene expression is highly stochastic [14]. Thus, there has been considerable effort at devising methods to infer parameters of auto-regulatory gene regulatory networks from noisy time course data using the chemical master equation (the discrete state and continuous time stochastic description of reaction kinetics [15]) or one of its numerous approximations [16–22]. These studies can be distinguished according to the type of kinetics used to describe auto-regulatory networks (mass-action or non-mass-action) and by the choice of method used to perform parameter inference (approximate Bayesian computation, Markov chain Monte Carlo (MCMC) algorithms and maximum likelihood methods).

Studies assuming mass-action kinetics, such as [16–19], describe the interactions of DNA, mRNA and protein using the first- and second-order reactions while those using non-mass action kinetic models [20–22] employ Hill or logical functions to describe effective interactions between mRNA and protein without an explicit description of the DNA. Approximate Bayesian computing approaches perform exhaustive stochastic simulations using the stochastic simulation algorithm (SSA) [23] and accept parameter values if the differences between simulation and experimental data are sufficiently small [19,24,25]. These methods are asymptotically exact, but they suffer from poor computational efficiency due to the very large number of required SSA runs. Inference using the Finite State Projection (FSP) algorithm is usually more efficient than that using the SSA; however, this is limited to small reaction networks [26]. A different approach, which is relatively more computationally efficient, involves approximating the likelihood (by approximating the chemical master equation) and then using a random walk scheme (MCMC), to explore parameter space and thus to finally obtain the posterior distributions of parameters [16,18,21,22,27,28]. The most common approximations used are the linear-noise approximation (LNA) and the two-moment approximation (2MA), presumably because these are the simplest and most well-known approximations of the chemical master equation in the literature of stochastic chemical kinetics [29]. A third (non-Bayesian) approach is typically the most computationally efficient of the approaches mentioned thus far and involves a direct maximization of the approximate likelihood using numerical optimization techniques [17,30,31]. We collectively label the aforementioned MCMC and maximum likelihood methods under the umbrella of *moment-based* inference because they involve solving a closed set of ordinary differential equations for the approximate moments. We emphasize that approximations are necessary because the chemical master equation can rarely be solved for all times when the reaction system has bimolecular reactions [29], and such reactions are very common *in vivo*, e.g. the protein–DNA binding reaction in an auto-regulatory transcriptional feedback loop.

All auto-regulatory networks have two properties in common: (i) they are typically very noisy particularly as proteins are produced in short bursts due to translational bursting [32] and (ii) they all have at least one protein–DNA bimolecular reaction which controls the strength of feedback. Unfortunately, common approximation methods, such as the LNA and the 2MA, are valid in the limit of small noise [33] and the error between their predictions and the exact solution of the chemical master equation increases with the size of bimolecular rate constants [29,33,34]. The question of how accurate the parameter estimates are is thus a pressing one and it has not been addressed properly because published studies to date have focused on method development and only verified the method’s accuracy on a few parameter sets. In this article, we fill this gap in the literature by performing an exhaustive systematic analysis to understand the factors affecting the accuracy of parameter prediction in auto-regulatory transcriptional feedback loops using moment-based inference methods. In particular, we study how the accuracy of parameter estimation, using both MCMC and maximum-likelihood methods, varies across large swaths of parameter space and how the accuracy is affected by the number of time points of the observed data, the number of cells from which data are collected, the highest order of the moments used for inference and the choice of moment-closure method used to approximate the likelihood. Our results show that for cases where large bursts in protein production are evident and/or where strong feedback is suspected, approximation of the likelihood using the LNA and 2MA leads to large errors in the parameter estimates; this can be avoided by the use of more sophisticated moment-closure techniques.

## 2. Methods

### 2.1. Model of an auto-regulatory transcriptional feedback loop

**Figure 1.**
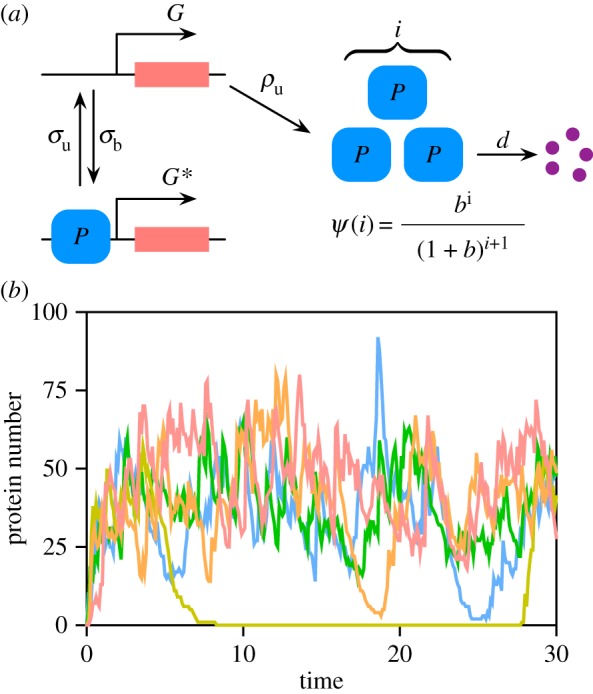
(*a*) Schematic of the negative auto-regulatory transcriptional feedback loop and the parameters to be inferred: the protein production rate *ρ*_u_, the mean protein burst size *b*, the degradation rate *d*, and the promoter switching rates *σ*_b_ and *σ*_u_. The burst size distribution is geometric and given by *ψ*(*i*) (see text for justification). (*b*) Five (independent) single cell trajectories generated using the SSA for the parameter set: *ρ*_u_ = 13, *b* = 3, *d* = 1, *σ*_b_ = 0.001 and *σ*_u_ = 0.1.

The auto-regulatory (repressive) genetic feedback loop which is the centre of this study is shown in figure 1*a*. When a gene is in the ON state (*G*), proteins are produced and subsequently degraded via a first-order reaction. The protein can bind to the gene and turn it OFF (denoted as the state G*); in this state, the protein can only be degraded. The proteins are produced in bursts with a mean burst size *b*. Note that the latter is the mean number of proteins produced per mRNA during its lifetime. The burst size distribution is chosen to be geometric; this distribution was previously derived for the common case of fast mRNA decay (translational bursting) [35] and has been also verified experimentally [32]. Hence, while mRNA is not explicitly described in our model, its effects are implicitly described through the protein burst size distribution. Note that transcriptional bursting (bursts of mRNA occurring when the promoter spends a long time in the OFF state) is also implicit by the same reasoning.

If the cells are identical, then the model has five parameters to be estimated: *ρ*_*u*_ (rate of protein production), *d* (rate of protein degradation), *b* (mean protein burst size), *σ*_*b*_ (the binding rate of protein to gene in the ON state) and *σ*_*u*_ (the rate at which the gene switches from OFF to ON). If the cells are non-identical, then we assume a lognormal distribution in *ρ*_u_ (see §3.2 for justification and detailed discussion of cellular heterogeneity) and there are six parameters to be determined: the mean and standard deviation of *ρ*_u_, *b*, *d*, *σ*_b_ and *σ*_u_.

### 2.2. Synthetic data

Consider an experimental set-up where the number of molecules of a certain protein is measured for *N* cells at *L* different time points. This is usually done by using an empirical formula to convert the fluorescence of a tagged protein in a cell to the number of molecules in that cell. If *x*_*i*_(*t*_*l*_) is the number of proteins in cell *i* at the *l*th time point *t*_*l*_, then we can calculate the set of *k*th central moment measurements, i.e. ${\hat{\mu }}_{k}=\{{\hat{\mu }}_{k}(t_{1}),\ldots ,{\hat{\mu }}_{k}(t_{L})\}$ where:

2.1 $$\begin{array}{rl} {\hat{\mu }}_{1}(t_{l}) & =\frac{1}{N}\sum_{i=1}^{N}x_{i}(t_{l})\;\text{and}\\ \;{\hat{\mu }}_{k}(t_{l}) & =\frac{1}{N}\sum_{i=1}^{N}{\left(x_{i}(t_{l})-{\hat{\mu }}_{1}(t_{l})\right)}^{k},\;k>1. \end{array}$$

The experimental set-up can be of two types: (i) fluorescence from the same *N* cells is measured at each time point, i.e. single cell data where individual cells can be tracked or (ii) population snapshot data whereby *N* cells are randomly selected from a much larger cell population such that the chances that the same cell is measured at different time points are negligible, e.g. flow cytometry. For both cases, we will make the simplifying assumption that there is no correlation between fluorescence measurements at any two different points in time. This assumption is naturally enforced when collecting population snapshot data. For single cell data, this assumption holds provided the interval between consecutive time points is much larger than the autocorrelation time of protein fluctuations.

We simulate an experiment and generate synthetic data using the SSA. The time series data for the auto-regulatory circuit shown in figure 1*a* (the number of proteins sampled at a number of equidistant time points) are generated for a certain set of values of the parameters using the SSA. Specifically the algorithm simulates the following set of reactions:

2.2 $$G\overset{\rho_{u}}{⟶}G+mP,\;G+P\overset{\sigma_{b}}{⟶}G^{*},\;G^{*}\overset{\sigma_{u}}{⟶}G\;\text{and}\;P\overset{d}{⟶}\emptyset ,$$

where *m* is a discrete random variable sampled from the geometric distribution *ψ*(*m*) = *b*^*m*^/(1 + *b*)^*m*+1^. The initial condition is zero proteins in state *G*. Each realization of the SSA simulates temporal data measured from a single cell (figure 1*b* shows typical single cell trajectories). For each time point, we then compute the moments of the molecule numbers across the population of cells using equation (2.1). This is the data input to the inference methods which are described next.

### 2.3. Bayesian inference

We will assume that the number of cells in our experiments is quite large such that by the central limit theorem the sample moments are approximately Gaussian distributed. This assumption is readily fulfilled in flow cytometry experiments where measurements of tens of thousands of cells or more [27,36] are routine. It is less clear if the assumption is valid for microfluidic set-ups which collect single cell data and which typically can at most sample of the order of a thousand cells [37].

The simplest method of inference would involve using only mean data but unfortunately for our auto-regulatory circuit this method does not enable the identification of all parameters—this is since *ρ*_*u*_ and *b* appear as a product in the rate equations for the mean concentrations (see equation (B 3) in appendix B) which makes their individual estimation impossible (the implicit reason is that the effective mean rate of protein production at any time is *ρ*_u_*b*). Hence at least the mean and variance of protein numbers at each time point are needed to identify all parameters. Now it is known that the covariance between the sample mean and the sample variance at each time point tends to zero as the sample size increases [38]. Hence for large cell numbers, the likelihood that at time point *t*_*l*_ we measure the first and second central moments $\left\{{\hat{\mu }}_{1}(t_{l}),{\hat{\mu }}_{2}(t_{l})\right\}$ given the parameter vector *θ*, can approximately be written as the product of two Gaussians, one for the mean and one for the variance:

2.3 $$L_{l}({\hat{\mu }}_{1}(t_{l}),{\hat{\mu }}_{2}(t_{l})|\theta )=\prod_{k=1}^{2}\frac{1}{\sqrt{2\pi \sigma_{k}^{2}(t_{l})}}\exp\left(-\frac{{\left({\hat{\mu }}_{k}(t_{l})-{\tilde{\mu }}_{k}(t_{l},\theta )\right)}^{2}}{2\sigma_{k}^{2}(t_{l})}\right),$$

where the variance $\sigma_{k}^{2}(t_{l})$ is related to moment measurements by the equations:

2.4 $$\begin{array}{r} \sigma_{1}^{2}(t_{l})=\frac{1}{N}{\hat{\mu }}_{2}^{2}(t_{l})\;\text{and}\;\sigma_{2}^{2}(t_{l})=\frac{1}{N}\left({\hat{\mu }}_{4}(t_{l})-\frac{N-3}{N-1}{\hat{\mu }}_{2}^{2}(t_{l})\right), \end{array}$$

and ${\tilde{u}}_{k}(t_{l},\theta )$ is the *k*th moment at time *t*_*l*_ as predicted by the chemical master equation given the parameter vector *θ*. Since most master equations cannot be solved when there are protein–DNA binding reactions [29], an approximation of the master equation is necessary to calculate the likelihood above. Zechner *et al.* [27] used the 2MA whereby one obtains closed approximate equations for the first two moments from the chemical master equation by assuming that the third-order cumulants are zero [33,39]. However, generally the approximation method used can be any type of moment-closure method (see next section).

Due to the independence of fluorescence measurements at any two different points in time, it then follows that the likelihood that we measure the moment vectors ${\hat{\mu }}_{1},{\hat{\mu }}_{2}$ (the first two moments measured at *L* time points) given the parameter vector *θ*, is as follows:

2.5 $$L({\hat{\mu }}_{1},{\hat{\mu }}_{2}|\theta )=\prod_{k=1}^{2}\prod_{l=1}^{L}\frac{1}{\sqrt{2\pi \sigma_{k}^{2}(t_{l})}}\exp\left(-\frac{({\hat{\mu }}_{k}(t_{l})-{\tilde{\mu }}_{k}{\left(t_{l},\theta )\right)}^{2}}{2\sigma_{k}^{2}(t_{l})}\right).$$

Thus within a Bayesian framework the posterior distribution of the parameter vector *θ* is given by

2.6 $$p(\theta |{\hat{\mu }}_{1},{\hat{\mu }}_{2})\propto L({\hat{\mu }}_{1},{\hat{\mu }}_{2}|\theta )p(\theta ),$$

where *p*(*θ*) is the prior distribution on *θ*. A parameter search can then be performed to maximize the parameter posterior using an adaptive Metropolis–Hastings MCMC sampler (see appendix A for a description of the algorithm, choice of prior and proposal distributions, burnin time, etc.). MCMC samples converge in distribution to the posterior, and as such any statistics computed using a finite sample (after the burnin time) is an approximation to the posterior. We define the highest mode of the posterior distribution (the *maximum a posteriori*, MAP) to be the parameter estimate and the width of the distribution is a measure of uncertainty. The use of an adaptive sampler prevents the chain getting easily stuck by adapting to the global covariance of the posterior distribution. The MCMC was coded in the Julia language [40] and its typical runtime (to achieve convergence of the chain) for the applications discussed in this paper was many hours, in some cases as high as 20 h (all simulations run on a single core of an Intel^®^ Xeon^®^ Silver 4114 CPU @ 2.20 GHz). The $\hat{R}$ ratio [41] was very close to 1 for times larger than the burn-in time which is a strong indicator of chain convergence.

We note that this method can be easily extended to include information about higher-order moments than two. For example, if we wished to use the first three central moments of the protein number data for inference, then equation (2.5) would be replaced by

2.7 $$L({\hat{\mu }}_{1},{\hat{\mu }}_{2},{\hat{\mu }}_{3}|\theta )=\prod_{k=1}^{3}\prod_{l=1}^{L}\frac{1}{\sqrt{2\pi \sigma_{k}^{2}(t_{l})}}\exp\left(-\frac{({\hat{\mu }}_{k}(t_{l})-{\tilde{\mu }}_{k}{\left(t_{l},\theta )\right)}^{2}}{2\sigma_{k}^{2}(t_{l})}\right),$$

where the variance $\sigma_{k}^{2}(t_{l})$ is related to moment measurements by equation (2.4) and one further equation

2.8 $$\sigma_{3}^{2}(t_{l})≃\frac{1}{N}({\hat{\mu }}_{6}(t_{l})-{\hat{\mu }}_{3}^{2}(t_{l})).$$

### 2.4. Maximum-likelihood estimator

An alternative frequentist method of estimation involves finding the parameter vector that maximizes the likelihood. It is immediately clear from the form of equation (2.5) that this is tantamount to minimizing the negative logarithm of the likelihood. To be specific, the parameter vector is found by solving the optimization problem:

2.9 $$\min_{\theta }\sum_{k=1}^{2}\sum_{l=1}^{L}\frac{{\left({\hat{\mu }}_{k}(t_{l})-{\tilde{\mu }}_{k}(t_{l},\theta )\right)}^{2}}{\sigma_{k}^{2}(t_{l})}.$$

This is the maximum-likelihood estimator (MLE) that we use throughout this paper. Note that the pre-factors are neglected due to their constant values. The positivity constraint on the parameter values can be easily handled by the ln − exp transformation. Specifically, equation (2.9) is equivalent to:

$$\min_{\theta_{e}}\sum_{k=1}^{2}\sum_{l=1}^{L}\frac{({\hat{\mu }}_{k}(t_{l})-{\tilde{\mu }}_{k}{\left(t_{l},\exp(\theta_{e}))\right)}^{2}}{\sigma_{k}^{2}(t_{l})},$$

allowing *θ*_e_ to be the optimization variables over the entire real space, and the actual parameters can then be deduced from exp (*θ*_e_). Of course, this estimator can also be extended to include information about higher-order moments than two by changing the upper limit of the sum over *k*. The MLE estimator here used can be seen as a special case of the generalized method of moments estimator used in [42].

Since the variances $\sigma_{1}^{2}(t_{l})$ and $\sigma_{2}^{2}(t_{l})$ converge to 0 when the number of cells *N* tends to infinity, the normal distributions in the likelihood equation (2.5) turn to Delta functions which are only non-zero for ${\hat{\mu }}_{k}(t_{l})={\tilde{\mu }}_{k}(t_{l},\theta )$. Thus it follows that in the infinite cell number limit, the MAP estimate from MCMC will be equal to the value of *θ* which minimizes the mismatch between the predictions and measurements of moments, which is the same value obtained from the MLE equation (2.9). This is of course only true if the support of the prior distribution is wide enough.

MLE is computationally very efficient compared to moment-based Bayesian inference using an adaptive MCMC sampler; this is its main advantage. A main difference from Bayesian inference is that it leads to a point-wise estimate of the model parameters (rather than a posterior distribution). The MLE was computed using an adaptive differential evolution Algorithm [43,44] implemented in the Julia language [45]. This leads to an efficient global numerical optimization with a typical runtime under a minute for the applications discussed in this paper.

### 2.5. Computation of error in parameter estimates

The set of synthetic moments generated by the SSA is the input to the MLE and MCMC algorithms described in the previous sections which subsequently output predictions for the parameter values. We then compute two types of fractional errors for each parameter *θ*_*i*_:

2.10 $${\text{FE}}_{\mathrm{MLE}-\mathrm{MAP}}=\frac{\left|\theta_{i,\mathrm{MLE}}-\theta_{i,\mathrm{MAP}}\right|}{\theta_{i,\mathrm{MAP}}},$$

2.11 $${\text{FE}}_{\mathrm{MAP}-\mathrm{True}}=\frac{\left|\theta_{i,\mathrm{MAP}}-\theta_{i,\mathrm{True}}\right|}{\theta_{i,\mathrm{True}}}$$

2.12 $$\text{and}\;\;\;{\text{FE}}_{\mathrm{MLE}-\mathrm{True}}=\frac{\left|\theta_{i,\mathrm{MLE}}-\theta_{i,\mathrm{True}}\right|}{\theta_{i,\mathrm{True}}}.$$

The first error quantifies the difference between the MLE and the mode of the MCMC-derived posterior (the MAP estimate), while the second and third errors quantify the error between the MAP estimate (or the MLE estimate) and the true parameter value, i.e. the parameter values input into the SSA and used to generate the synthetic data.

### 2.6. Choices for the moment-closure approximation method

Since the chemical master equation of the feedback loop can only be solved in steady state [46], the likelihoods need to be approximated by a moment-closure method. There are a wide variety of such methods [47], each with their own advantages. We shall consider six types of approximations: LNA [15], the three moment approximation (3MA) [33], derivative matching (DM) [48], conditional derivative matching (CDM) [49], conditional Gaussian approximation (CG) [49] and the linear-mapping approximation (LMA) [50]. The LNA was described in the Introduction. The 3MA is an elaboration of the 2MA explained earlier; while in the latter we assume the third cumulant is zero, in the former we assume that the fourth cumulant is zero. The 3MA gives a closed set of equations for the first three moments and is a more accurate approximation of the chemical master equation than the 2MA [33,51]. Hence, in this article, we use the 3MA instead of the more common 2MA. DM involves matching time derivatives of the exact (not closed) moment equations with that of the approximate (closed) moment equations at some initial time. CDM is a conditional version of DM, i.e. where DM is performed conditional on the state of the low abundance species, e.g. the promoter states. CG is a special case of the conditional method of moments developed earlier by Hasenauer *et al.* [52]; it can also be seen as a conditional version of the 2MA, again where the conditioning is on the promoter state. The LMA is a not a true moment-closure method in the usual sense of the word because it actually gives approximate expressions for the time-dependent probability distributions of a wide class of gene regulatory networks (which moment-closure methods cannot give). The LMA is based on an approximate mapping of the dynamics of a gene regulatory system with protein–DNA binding reactions to a system with no binding reactions. Appendices B and C contain the equations defining each of these closures for the auto-regulatory transcriptional feedback loop for the case of identical and non-identical cells, respectively.

## 3. Investigating the factors that influence the accuracy of inference

### 3.1. Inference from identical cells

In this section, we study the various factors that influence the accuracy of inference of the parameters of a negative auto-regulatory genetic feedback loop from synthetic data generated for a population of cells using the SSA (§2.2) where the inference is done using a Bayesian and a frequentist method (§§2.3 and 2.4, respectively). We systematically investigate the error in the parameter predictions as a function of all user-input variables: (i) the number of cells at each time point; (ii) the number of time points; (iii) the highest-order moment used for inference; (iv) the moment-closure method used for likelihood approximation.

*Testing the independence assumption of the likelihood function.* We first explicitly confirm the assumption behind our method of inference, namely that the sample mean and sample variance are independent at each time point such that we can write the likelihood as a product of likelihoods for each moment (see §2.3). We fix the parameters in the SSA to *ρ*_u_ = 13, *b* = 3, *d* = 1, *σ*_b_ = 0.001 and *σ*_u_ = 0.1, generate the synthetic data for a number of *N* cells at 30 time points (interval 1), compute the central moments using equation (2.1) and the correlation coefficient of sample mean and sample variance for time *t*_*l*_ using the following:

3.1 $$\rho ({\hat{\mu }}_{1}(t_{l}),{\hat{\mu }}_{2}(t_{l}))=\frac{\text{cov}({\hat{\mu }}_{1}(t_{l}),{\hat{\mu }}_{2}(t_{l}))}{\sigma_{1}(t_{l})\sigma_{2}(t_{l})}=\frac{{\hat{\mu }}_{3}(t_{l})}{N\sigma_{1}(t_{l})\sigma_{2}(t_{l})}.$$

**Figure 2.**
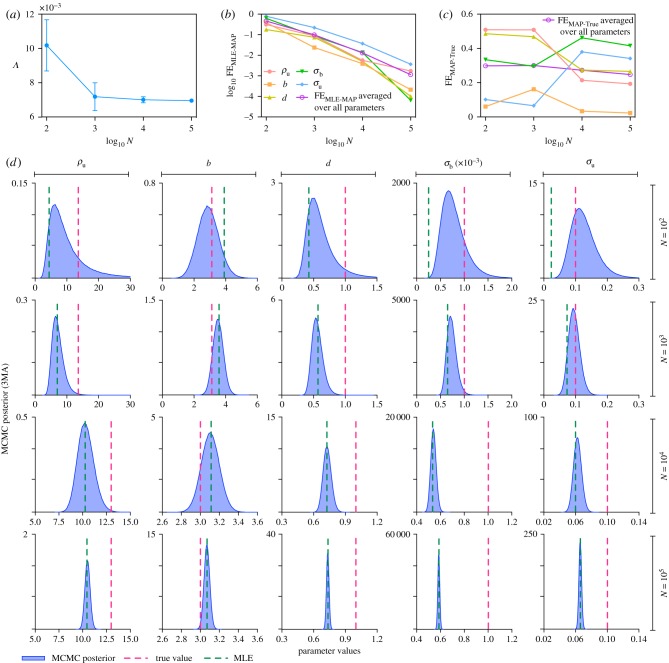
(*a*) Plot of the time-averaged correlation coefficient of the first two moments, **Λ**, versus the sample size (number of cells) *N*. The small values of **Λ** verify the assumption of independence of sample mean and sample variance in the likelihood equation (2.3). (b) Plot of the fractional error between the MLE and MAP estimate from MCMC equation (2.10) as a function of the sample size. The error decreases rapidly and is less than 1% for *N* = 10^5^, a common sample size. (*c*) Plot of the fractional error between the true value and MAP estimate from MCMC equation (2.11) as a function of the sample size. The error does not converge to zero as the sample size increases. This error is the systematic error which stems from the likelihood approximation by moment-closure and remains in the limit of infinite sample size. (*d*) Comparison of MCMC posterior distributions (blue), MAP estimates (mode of distribution), MLE (green dashed line) and true value (red dashed line) of the five parameters as a function of sample size. The data complements (*b*) and (*c*) and shows the convergence of the posterior to a value that is significantly different from the true parameter value. The parameters used for SSA to generate synthetic data are *ρ*_u_ = 13, *b* = 3, *d* = 1, *σ*_b_ = 0.001 and *σ*_u_ = 0.1, and the underlying moment equations are closed by means of 3MA. The sample mean and sample variance of protein numbers are measured at discrete time *t* = 1, 2, …, 30.

Note that the last step uses an exact result for the covariance of sample mean and sample variance derived in [38]. In figure 2*a*, we show $\rho ({\hat{\mu }}_{1}(t_{l}),{\hat{\mu }}_{2}(t_{l}))$ averaged over all 30 time points (denoted as *Λ*) as a function of *N*. The very small value of the time-averaged correlation coefficient of sample mean and sample variance is practically negligible for populations with more than a hundred cells and hence the assumption of independence of sample mean and sample variance in our inference methods holds. This was found to be the case for all parameter values explored in this study.

*Quantifying the differences between the MLE and MAP estimate as a function of *N*.* We now fix the moment-closure method of approximating the likelihood to be the 3MA and fix the number of time points to 30 (with interval 1). In figure 2*b*, we plot FE_MLE−MAP_ (see equation (2.10)) as a function of *N* which quantifies the difference in the parameters obtained using the MLE and the MAP estimate of MCMC. The fractional errors decrease rapidly with increasing *N* showing rapid convergence of the two estimates. The number of cells in flow cytometry measurements (we shall refer to this as the sample size from now on) tends to be much larger than 10^4^ and hence the difference between the two estimates (for all five parameters) is less than 3%. In figure 2*d*, we show the corresponding posterior distributions obtained from MCMC for each of the five parameters as a function of *N*, while the vertical green dashed line shows the MLE. Note that these results do, of course, depend on the choice of prior distribution but are independent of the particular choice, we always found that the fractional error between the MLE and MAP estimates decreases rapidly with *N* (this is, of course, expected as the amount of informative observations increases, the effect of the prior is diluted). The very small differences between the two estimators make a strong case for the use of the MLE rather than the MCMC method for typical flow cytometry sample sizes since the computational time of the former is at most a few minutes, while of the latter is many hours.

*Quantifying the differences between the inferred and true parameter values as a function of *N*.* In figure 2*c*, we plot FE_MAP−True_ (see equation (2.11)) as a function of *N* which quantifies the difference between the MAP estimate of the parameter and the true value of the parameter. This figure clearly shows that the percentage error does not significantly decrease with *N* and can be as high as 40% for typical flow cytometry sample sizes. Now the sampling error due to the finite cell number *N* and the error due to assuming independence of sample mean and sample variance rapidly go to zero as *N* → ∞. The only error remaining in this limit is the systematic error which is the error due to likelihood approximation by the moment-closure method. Hence, figure 2*c* shows that the systematic error due to likelihood approximation by the 3MA by far dominates the other errors. The differences between the MAP estimate and the true value (red vertical line) can be better appreciated in figure 2*d* where we plot the posteriors of the parameter distributions as a function of *N*. In particular, the case *N* = 10^5^ (last row of figures in figure 2*d*) is remarkable since the red vertical line (the true value) is way off from the narrowly peaked (converged) posterior. Note that since the differences between MLE and the MAP estimate are very small (figure 2*b*), the error computed between the MLE and the true value, FE_MLE−True_, is very similar to that reported in figure 2*c* for FE_MAP−True_.

**Figure 3.**
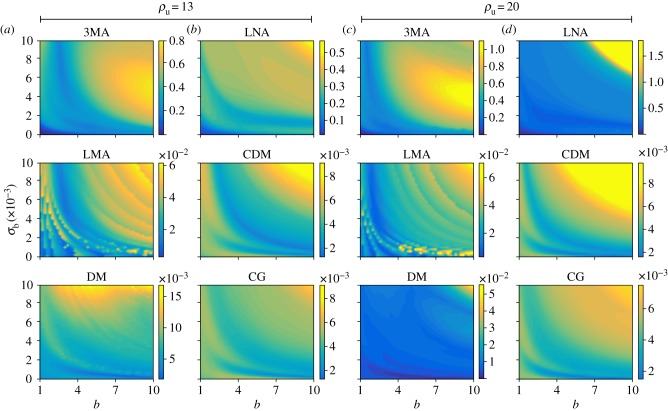
Heat map showing the systematic error in the MLE due to likelihood approximation in the limit of infinite sample size by six different moment-closures as a function of the protein–DNA binding rate *σ*_b_, the mean burst *b* and the protein production rate *ρ*_u_. The closures are described in §2.6. The error in all methods tends to increase with *σ*_b_ and *b*. The maximum error is of order 1 for the 3MA and LNA, while it is of order 10^−2^ for the LMA, CDM, DM and CG. The fixed parameter values are *d* = 1 and *σ*_u_ = 0.1, while the number of time points is *L* = 30. The mean burst size *b* was varied in the range 1–10 in agreement with published experimental values [8,60]. The sample size is effectively infinite because the synthetic data are generated using FSP (see main text for discussion).

*Quantifying the systematic error in the inferred parameter values as a function of the type of moment-closure approximation for the likelihood.* We have previously found that the systematic error was very large using the 3MA. Next we investigate how this error varies with the choice of moment-closure approximation. Since it is computationally unfeasible to generate a very large number of cell samples using the SSA, for this study we use the FSP algorithm [53]) to directly obtain the time-dependent probability distribution of the genetic feedback loop, from which we calculate the moments for 30 time points (interval one). Because we truncated the FSP to a very large protein number compared to the mean protein number, the results obtained from FSP are practically the same as the exact solution of the master equation, i.e. the limit *N* → ∞ of the SSA. In particular, by comparing the mean and variance from FSP with that from SSA, for various parameter sets, we estimated that the relative error in FSP’s moments is less than 1% for all times. We then use the moments of the probability distribution at the 30 time points to generate the MLE of the five parameters. These and the true parameter values are used to calculate the fractional error for each parameter using equation (2.12). In figure 3, we show a heat map of the fractional error averaged over all five parameters as a function *σ*_*b*_, *b* and *ρ*_*u*_ for the six different types of moment-closure approximations mentioned in the §2.6. The heat map shows that the systematic error increases rapidly with increasing rate of protein–DNA binding *σ*_*b*_ and with increasing mean burst size *b* (there is only a weak dependence on the translation rate of proteins in the ON state *ρ*_*u*_). This dependence is to be expected since (i) *σ*_*b*_ controls the strength of the only bimolecular reaction in the feedback loop and we know that it is the presence of this reaction which necessitates the use of moment-closure approximation (from the master equation of a system with only zero or first-order reactions, one can derive a closed set of moment equations and hence no approximation is necessary in this case [29]). (ii) *b* controls the size of protein number fluctuations and we know that most approximations are valid for small noise only [29,33]. Note that the maximum systematic error using the 3MA and the LNA is of order 1, while the maximum systematic error using the LMA, CDM, DM or CG is of order 10^−2^. The 2MA (the lesser accurate version of the 3MA) and the LNA have been the methods of choice for inference in the literature, presumably because of their simplicity. Hence our results make a strong case for the use of the more sophisticated LMA, CDM, DM or CG for cases where large bursts in protein production are evident and/or where strong feedback is suspected.

**Figure 4.**
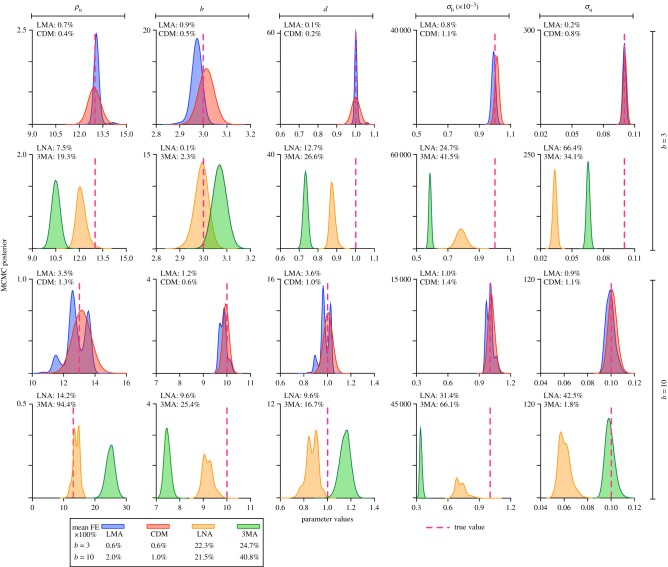
Comparison of MCMC posterior distributions for each of the five parameters using likelihood approximation based on four types of moment-closure: 3MA, LNA, CDM and LMA for two different values of the mean burst size *b*. The MAP estimate of the LMA and CDM posteriors is in all cases close to the true value (red dashed vertical line). By contrast, the MAP estimate of the 3MA and LNA is way off the true value. The percentage error of each parameter (computed using equation (2.11) and multiply by 100) is indicated in the top left corner of the plots, whereas the percentage error averaged over all parameters is summarized in the legend. Synthetic data are generated using the SSA for a sample size of 10^5^ and number of time points *L* = 30. The parameters are *ρ*_u_ = 13, *d* = 1, *σ*_b_ = 0.001, *σ*_u_ = 0.1, *b* = 3 or 10.

We further corroborated the results in figure 3 by generating synthetic SSA data for two points in parameter space, *ρ*_*u*_ = 13, *d* = 1, *σ*_*b*_ = 0.001, *σ*_*u*_ = 0.1 and *b* = 3 or *b* = 10 with *N* = 10^5^ cells and 30 time points and then estimating the parameters using MLE and MCMC with the likelihood approximated using 3MA, LNA, LMA and CDM (the other two types of moment-closure, DM and CG, give very similar results to the LMA and CDM and hence we have not included them). The results are shown in figure 4. The LMA and CDM percentage errors (averaged over all five parameters) are in the range 0.6–2%, while the 3MA and LNA errors are in the range 22–41% which is in good agreement with the heat map in figure 3 generated with MLE using FSP synthetic data. This figure, however, provides additional important information: it shows the error for each parameter and the posterior distributions obtained from MCMC. The error in the protein–DNA binding rate *σ*_*b*_ is the largest or the second largest error among the five parameters when the LNA and 3MA are used to approximate the likelihood. It is visually clear that the posteriors generated using the LMA and CDM (blue and red distributions, respectively, in the first and third rows of figure 4) are centred or almost centred on the true parameter value (red vertical line)—this is obviously not true for the posteriors generated using the LNA and 3MA (yellow and green distributions, respectively, in the second and fourth rows of figure 4). However, the posteriors from the LMA and CDM are not necessarily narrower than those from the LNA and 3MA and hence the choice of moment closure scheme does not appear to significantly impact the uncertainty in the MAP estimate. In tables 1 and 2, we compare the MAP estimate of MCMC in figure 4 with the MLE for the same synthetic SSA data as well as with the MLE using synthetic FSP data (reported in figure 3). All three are in good agreement for the four moment-closures tested, thus providing another verification of the superiority of LMA/CDM over LNA/3MA for moment-based inference.

**Table 1.** Burst size *b* = 3.

moment-closure type inference method		LMA			CDM			LNA			3MA			
		MCMC	MLE	MLE (FSP)	MCMC	MLE	MLE (FSP)	MCMC	MLE	MLE (FSP)	MCMC	MLE	MLE (FSP)	True
parameter estimate	*ρ*_u_	13.09	13.00	13.08	12.94	12.95	13.10	12.03	12.06	12.38	10.49	10.51	10.64	13
	*b*	2.97	3.00	2.97	3.01	3.01	3.00	3.00	3.00	2.97	3.07	3.07	3.05	3
	*d*	1.00	1.00	1.00	1.01	1.00	1.01	0.87	0.88	0.89	0.73	0.73	0.74	1
	*σ*_b_( × 10^−3^)	0.99	1.01	0.98	1.00	1.01	1.00	0.75	0.75	0.74	0.58	0.58	0.58	1
	*σ*_u_	0.10	0.10	0.10	0.10	0.10	0.10	0.03	0.03	0.03	0.07	0.07	0.06	0.1
mean FE ×100%		0.55%	0.33%	0.87%	0.60%	0.58%	0.44%	22.29%	22.28%	22.14%	24.77%	24.80%	24.66%	0

**Table 2.** Burst size *b* = 10.

moment-closure type inference method		LMA			CDM			LNA			3MA			
		MCMC	MLE	MLE (FSP)	MCMC	MLE	MLE (FSP)	MCMC	MLE	MLE (FSP)	MCMC	MLE	MLE (FSP)	True
parameter estimate	*ρ*_u_	12.55	12.49	12.40	13.16	13.23	12.99	14.85	14.96	14.33	25.27	25.54	23.86	13
	*b*	9.88	9.95	9.98	9.94	9.94	10.04	9.04	9.01	9.16	7.46	7.41	7.59	10
	*d*	0.96	0.96	0.96	1.01	1.01	1.00	0.90	0.91	0.89	1.17	1.17	1.12	1
	*σ*_b_( × 10^−3^)	1.01	1.01	1.00	1.01	1.01	1.00	0.69	0.68	0.69	0.34	0.34	0.34	1
	*σ*_u_	0.10	0.10	0.10	0.10	0.10	0.10	0.06	0.06	0.06	0.10	0.10	0.10	0.1
mean FE ×100%		2.01%	2.12%	1.98%	1.07%	1.01%	0.17%	21.47%	21.91%	20.73%	40.88%	41.80%	37.10%	0

In the electronic supplementary material, we also demonstrate the accuracy of distribution reconstruction from inferred parameters, the robustness of the MLE estimates to external noise and the convergence of the MCMC chain. A short description of each follows. In electronic supplementary material figure S1, we reconstruct the time-dependent distribution of molecule numbers (using FSP) based on 3MA and CDM inferred kinetic parameters reported in table 1. We find that both methods lead to a distribution that is visually close to that generated using the true parameter values, with the accuracy being highest for the CDM-reconstructed distribution which is virtually indistinguishable from the true distribution. We have also tested the robustness of the MLE inference method to noise added to the measured moments; this additional noise mimics sources of noise other than intrinsic noise inherent in the synthetic SSA data. In electronic supplementary material, figure S2, we show that the fractional error averaged over all parameters increases linearly with the size of added noise. In electronic supplementary material, figure S3, we plot the Gelman–Rubin $\hat{R}$ ratio as a function of the number of iterations of the MCMC chain where the likelihood is approximated using the LMA moment equations—the ratio quickly tends to 1 after the burn-in time demonstrating chain convergence. Note that the same quick convergence is seen for all MCMC results reported in this article.

**Figure 5.**
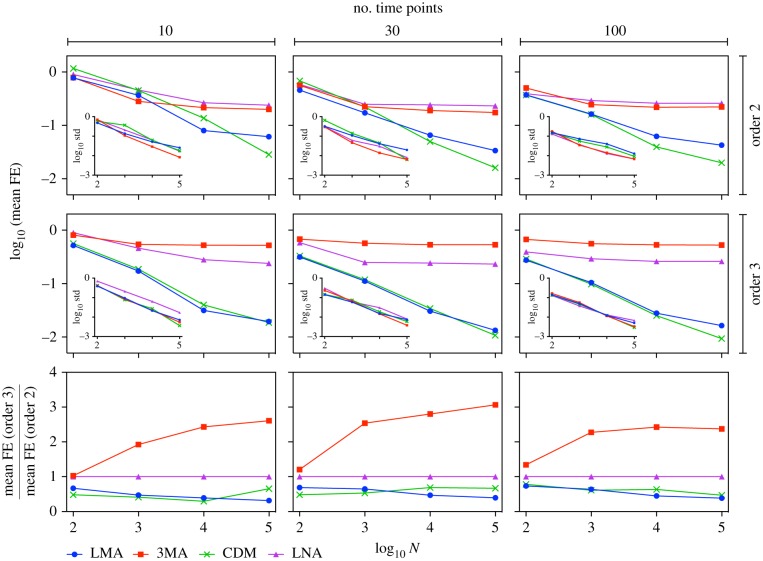
Influence of the number of time points, sample size, choice of moment-closure and the highest order of measured moments on the mean fractional error of parameter estimates computed using the MLE. Each point in the figure is calculated from 10 independent samples of synthetic data generated using the SSA. The insets show the corresponding standard deviation. The first and second row of figures show the mean fractional error over parameters using a likelihood informed by the first two sample moments, equation (2.5) and a likelihood informed by the first three sample moments, equation (2.7), respectively. The third row of figures shows the ratio of the mean fractional error using the two aforementioned likelihood approximations. The number of time points has a minor influence on the error, while the other factors (choice of moment-closure, sample size, highest order of measured moments) have a much larger effect on the error. As the highest order of measured moments is changed from 2 to 3, the LNA’s accuracy remains the same, the 3MA’s accuracy becomes worse, while the LMA and CDM’s accuracy is significantly improved (see main text for discussion). The parameters used are *ρ*_u_ = 13, *b* = 5, *d* = 1, *σ*_b_ = 0.001 and *σ*_u_ = 0.1.

*Quantifying the differences between the inferred and true parameter values as a function of the number of time points *L* and the highest-order moment used for inference.* Thus far, we have fixed the number of time points to *L* = 30 and the highest-order moment used to two. Now we relax both of these. In figure 5, we show the fractional error averaged over the five parameters computed using the MLE as a function of the cell numbers *N* for a total number of time points *L* = 10, 30, 100; the first and second row of figures use two and three as the highest-moment order, respectively. Data are shown for four types of moment-closure approximations: 3MA, LNA, CDM and LMA. In all cases, the parameter set is fixed to *ρ*_u_ = 13, *b* = 5, *d* = 1, *σ*_b_ = 0.001 and *σ*_u_ = 0.1. There are two main observations to be made: (i) the increase in the number of time points does not significantly change the mean fractional error; and (ii) the inclusion of measurements of third-order central moment improves the inference using the LMA and CDM, but makes the inference using the 3MA worse (see the third row of figures in figure 5). The LNA is insensitive to the inclusion, because the third-order central moments are always zero as per the underlying assumption of a Gaussian distribution. This analysis shows that the mean fractional error using the MLE depends strongly on the choice of moment-closure approximation and on the sample size *N*, less strongly on the highest-order moment used for inference and weakly on the number of time points. Results using the MAP estimate of MCMC lead to very similar results.

### 3.2. Inference from non-identical cells

**Figure 6.**
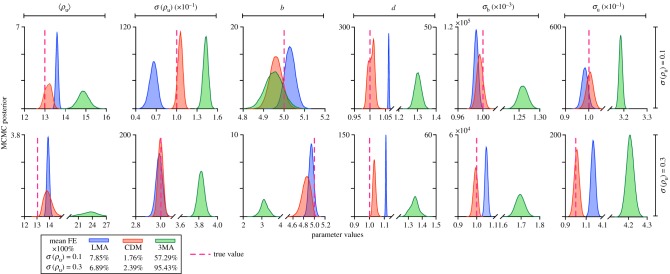
Comparison of MCMC posterior distributions for each of the six parameters of an auto-regulatory transcriptional feedback loop in a population of non-identical cells and as a function of the moment-closure type. The cells have identical parameters except for the protein production rate *ρ*_u_ which is chosen to be a lognormal with mean 〈*ρ*_u_〉 = 13 and standard deviation *σ*(*ρ*_u_) = 0.1 or 0.3. The percentage error averaged over all parameters is summarized in the legend. The synthetic data are generated using the SSA with sample size 10^6^ and 30 time points. The LMA and CDM have an average error which is at least an order of magnitude less than that of the 3MA.

Thus far, we have assumed inference from a population of identical cells, but, of course, this is an ideal which does not exist in nature. Variability between cells can be modelled by choosing rate constants to vary from one cell to another one. Generally, rate constants might even change with time in a single cell, but this is likely a secondary effect compared to cell-to-cell variation in the rate constants. In particular, previous experimental studies have shown that one of the major sources of gene expression variability in yeast is cell-to-cell variation in transcription factor expression [54]. In our model, the protein is the repressing transcription factor and its expression is controlled by the rate constant *ρ*_u_. Hence we choose this constant to vary from cell to cell, while the other rate constants are identical across cells. In agreement with previous studies, the distribution of *ρ*_u_ across cells is chosen to be lognormal [55]. Specifically we fix the parameter set to *b* = 5, *d* = 1, *σ*_b_ = 0.001, *σ*_u_ = 0.1 and *ρ*_u_ to be lognormally distributed (across cells) with mean 13 and standard deviation 0.1 or 0.3. Hence the parameters to be inferred are now six: the mean and standard deviation of *ρ*_u_, *b*, *d*, *σ*_u_ and *σ*_b_. Figure 6 shows the MCMC posterior distributions for these six parameters using the 3MA, LMA and CDM moment-closure approximations. The mean percentage error across all parameters is 57–95% using the 3MA, 7–8% using the LMA and 2% using the CDM. In comparison, the mean percentage error across all parameters is 25–41% using the 3MA, 0.6–2% using the LMA and 0.6–1% using the CDM for the case of identical cells (figure 4). The main conclusions to be drawn are as follows: (i) inference for non-identical cells leads to parameter predictions with significantly larger errors than that for identical cells; (ii) the LMA and CMD closure leads to much more accurate results than the 3MA—the CDM is particularly accurate and seems the best choice. We did not test the LNA, but since for identical cells the LNA and 3MA always fared very similar, we expect the same in this case too. In tables 3 and 4, we compare the MAP estimate of MCMC in figure 6 with the MLE using the same synthetic SSA data; as expected, we find the MLE and MAP estimates to agree very closely for all six parameters and using all moment-closure approximations.

**Table 3.** Extrinsic noise *σ*(*ρ*_u_) = 0.1.

moment-closure type inference method		LMA		CDM		3MA		
		MCMC	MLE	MCMC	MLE	MCMC	MLE	true
kinetic parameter estimate	〈*ρ*_u_〉	13.59	13.58	13.23	13.26	14.84	14.87	13
	*σ*(*ρ*_u_)	0.07	0.06	0.11	0.10	0.14	0.14	0.1
	*b*	5.03	5.03	4.96	4.95	4.96	4.95	5
	*d*	1.06	1.06	1.01	1.01	1.30	1.30	1
	*σ*_b_(×10^−3^)	0.99	0.99	0.99	0.99	1.26	1.26	1
	*σ*_u_	0.10	0.10	0.10	0.10	0.33	0.33	0.1
mean FE ×100%		7.85%	8.18%	1.76%	1.60%	57.29%	57.17%	0

**Table 4.** Extrinsic noise *σ*(*ρ*_u_) = 0.3.

moment-closure type inference method		LMA		CDM		3MA		
		MCMC	MLE	MCMC	MLE	MCMC	MLE	true
kinetic parameter estimate	〈*ρ*_u_〉	13.90	13.90	13.84	13.87	23.68	23.99	13
	*σ*(*ρ*_u_)	0.30	0.30	0.30	0.30	0.38	0.38	0.3
	*b*	4.94	4.94	4.86	4.85	3.11	3.09	5
	*d*	1.11	1.11	1.03	1.03	1.35	1.35	1
	*σ*_b_(×10^−3^)	1.05	1.05	0.99	0.99	1.69	1.69	1
	*σ*_u_	0.12	0.12	0.10	0.10	0.42	0.42	0.1
mean FE ×100%		6.89%	6.89%	2.39%	2.42%	95.43%	96.00%	0

## 4. Discussion and conclusion

In this article, we have reported the results of an exhaustive study of the factors influencing the accuracy of moment-based MCMC and MLE methods for an auto-regulatory transcriptional feedback loop. Using the Bayesian method devised in [27] and its corresponding MLE, we showed that using only the first two moments of synthetic protein data, the accuracy of parameter estimation for large sample sizes is largely controlled by the choice of moment-closure method used to approximate the likelihood. The errors were found to increase with the size of the protein–DNA binding rate, the mean protein burst size and the heterogeneity of transcription rate across the cell population. We showed that using only mean data is not sufficient to identify all parameters and that at least mean and variance are needed to perform such a task. Using more than two moments of synthetic protein data does not necessarily lead to better accuracy—in particular this does not affect the accuracy when using the LNA and makes the predictions using the 3MA even worse than using only two moments. For sample sizes larger than about a thousand, the number of time points used did not significantly affect the accuracy. By contrast, the choice of moment-closure method made a huge difference in the accuracy of parameter estimation. Our computational study of the error over large swaths of parameter space conclusively showed that the popular choice of LNA and of closures based on zero cumulant (such as the 3MA) leads to large maximum percentage errors in the estimated parameters in the approximate range 60–100%, while other types of closures such as the CDM boasted very small maximum errors of about 1%. Our study also confirms that for sample sizes typical of flow cytometry (tens of thousands of cells) MLE approaches are more favourable than Bayesian methods since both methods lead to virtually indistinguishable estimates (if the same likelihood approximation is used), but the computation of MLE takes a few minutes, while MCMC takes many hours. Of course, MCMC approaches have the additional advantage of estimating the uncertainty in the parameter estimates; however, this could also be computationally efficiently estimated using normal approximations to the posterior [41].

Our study was specifically for a negative auto-regulatory feedback transcriptional feedback loop which does not incorporate cooperativity in the protein–DNA binding reaction nor protein dimerization reactions, as some previous studies did. Incorporating both of these would lead to a higher degree of nonlinearity in the law of mass action (since both cooperativity and dimerization imply more second-order reactions in our model). Under such conditions, one would expect even larger errors from the prediction of moment-based inference methods than what we have found because moment-closure approximations naturally perform best for systems with weakly nonlinear mass action laws [29]. It would also be interesting to investigate (i) whether the results here found for negative feedback loops extend to positive feedback loops and (ii) how the present inference method can be extended for use with spatially extended data [56]. These are topics for a future study.

In few instances [16], some studies have used the chemical Fokker–Planck equation (CFPE) as a means to compute the approximate likelihood. This method cannot be used in our moment-based inference because the moment equations of the CFPE are not closed. However, given that it was proved in [33,57] that the 3MA is more accurate than the CFPE (in the limit of large system sizes) and that the CFPE’s predictions for the protein distributions of auto-regulatory gene regulatory networks [58] can be very different from those of the chemical master equation, it appears highly likely that Bayesian inference methods based on the CFPE cannot outperform the LMA, CDM, DM and CG moment-based methods described in this article.

It remains to be seen whether particular moment-closures are more advantageous compared to others when one is interested in the more general problem of inferring both the network connectivity and the parameter values. However, given the large translational mean protein bursts measured *in vivo* (in the approximate range of 1 to 1000; see fig. 5*a* in [8]) and the rapid increase in estimation error with mean burst size that we identified in this study, it seems likely that new techniques (such as those based on the concept of convergent moments [59]) may be needed to ensure accurate inference of complex noisy gene regulatory networks with multiple interconnected feedback loops.

## Supplementary Material

Supplementary Information

## Data accessibility

The adaptive MCMC and MLE Julia code for producing the results for sample size *N* = 10^5^ in figure 1*d* can be found at https://github.com/edwardcao3026/MAP-MLE.

## Appendix A. Implementation of adaptive Markov chain Monte Carlo

Our initial exploration used a traditional non-adaptive Metropolis–Hastings algorithm (as in [27]) but we noted that this often resulted in the chain getting stuck for very long periods of time. This problem can be traced to the very narrow likelihoods at large sample sizes. Since it is well known that the choice of proposal distribution strongly affects the time taken for the chain to converge, we opted to instead use an adaptive MCMC which removes the need of a particular choice by automatically tuning the proposal distribution using the history of the process [61]. This led to convergence in a reasonable amount of time. The particular updating mechanism we used is a variant of algorithm 4 in [62]. The pseudocode of the adaptive MCMC is presented below.

1. Extract statistics ${\hat{\mu }}_{k}(t_{l})$ (sample mean and sample variance) and $\sigma_{k}^{2}(t_{l})$ (variance of sample mean and variance of sample variance) for *k* = 1, 2 for all time points using the formulae equations (2.1) and (2.4).
2. Select wide lognormal prior *p*(*θ*) in equation (2.6) and initialize $\theta^{\mathrm{old}}\in R^{J}$.
3. Select lognormal proposal distribution *q*(*θ*^new^|*θ*^old^) and initialize covariance matrix as identity matrix $C\leftarrow I\in R^{J\times J}$.
4. Initialize the adaptive parameters: (i) the starting point and termination point of adaptation *I*_1_ = 100 and *I*_2_ = 5 × 10^5^, respectively; (ii) the optimal acceptance rate opt (0.23 in this paper); (iii) learning gain *K* (100 in this paper); (iv) Robbins–Monro order *ɛ* ∈ (0, 1] (0.9 for this paper); (v) *s*_d_ is initially set equal to (2.38)^2^/*J*; (vi) parameter for preventing singularity *ε*_d_ = 2 × 10^−5^**I**.
  **(* Do Adaptive Metropolis–Hasting MCMC *)**
5. For *i* from 1 to Number_of_samplings
6.  Sample *u* from uniform distribution $U_{\left[0,1\right]}$;
7.  Sample *θ*^new^ from proposal distribution *q*(*θ*^new^|*θ*^old^);
   **(* Calculate Probability *)**
8.   Calculate moment predictions ${\tilde{\mu }}_{k}(t_{l},\theta )$ from moment equations for all *t*_*l*_ and *k* and for both *θ*^new^ and *θ*^old^;
9.   Calculate $p(\theta |{\hat{\mu }}_{1},\;\ldots ,\;{\hat{\mu }}_{n})$ as per equation (2.6) for both *θ*^new^ and *θ*^old^ in which $p({\hat{\mu }}_{k}(t_{l})|\theta )$ is approximated
  by using normal distribution equation (2.5);
10.  Calculate both *q*(*θ*^new^|*θ*^old^) and *q*(*θ*^old^|*θ*^new^);
11.  Calculate acceptance probability
  $$a=\min\left\{1,\frac{\;p(\theta^{\mathrm{new}}|{\hat{\mu }}_{1},\;\ldots ,\;{\hat{\mu }}_{n})q(\theta^{\mathrm{old}}|\theta^{\mathrm{new}})}{p(\theta^{\mathrm{old}}|{\hat{\mu }}_{1},\;\ldots ,\;{\hat{\mu }}_{n})q(\theta^{\mathrm{new}}|\theta^{\mathrm{old}})}\right\};$$
    **(* Update and Record Estimates *)**
12.  If *u* ≤ *a*
13.    *θ*^old^ ← *θ*^new^ and record *θ*^old^ in *θ*_r_;
14.    Set marker ind = 1;
15.  else
16.    Reject *θ*^new^;
17.    Set marker ind = 0;
18.  end
19.  **(* Adaptive Mechanism for Proposal Covariance Updating *)**
20.  Update *δ* according to *δ* = *i*^−ɛ^*K*(ind − opt);
21.  Update *s*_d_ according to *s*_d_ ← exp (ln(*s*_d_) + *δ*);
22.  **(* Adaptation initiates at the *I*_1_-th trial and terminates at *I*_2_-th trial *)**
23.  if *i* > *I*_1_ and *i* < *I*_2_
24.    $C\leftarrow s_{d}\text{cov}(\theta_{r}^{⊤})+s_{d}\epsilon_{d}$;
25.  end
26. end
27. Calculate the probability distribution of all the recordings of *θ*^old^ in the last 2/3 chain length—this is the posterior.
28. Pick the mode of the posterior for each parameter as the MAP estimate for that parameter.

**Remark 1 (early adaptation termination)**

In practice, we use the early-adaptation-termination adaptive MCMC in which the adaptation is terminated after 5 × 10^5^ runs after the acceptance *a* reaches steady state (the change on *a* is below the tolerance). The motivation of early termination on adaptation is to circumvent the ever-increasing computation demand on the calculation of covariance in step 24.

**Remark 2 (prior)**

The prior for system without extrinsic noise is selected as $LN(\mu_{\mathrm{pr}},\sigma_{\mathrm{pr}}),$ where

$$\begin{array}{rl} & \mu_{\mathrm{pr}}={\left[6,\;1,\;2,\;2\times 10^{-3},\;0.2\right]}^{⊤}\;\text{and}\\ & \;\sigma_{\mathrm{pr}}=\;\text{diag}{\left[1,\;1,\;0.5,\;0.5,\;0.5\right]}^{⊤} \end{array}$$

for *ρ*_u_, *b*, *d*, *σ*_b_ and *σ*_u_, respectively, whereas for extrinsic noise the parameters of the prior are chosen as

$$\begin{array}{rl} & \mu_{\mathrm{pr}}={\left[6,\;0.2,\;1,\;2,\;2\times 10^{-3},\;0.2\right]}^{⊤}\;\text{and}\\ & \;\sigma_{\mathrm{pr}}=\;\text{diag}{\left[1,\;0.5,\;1,\;0.5,\;0.5,\;0.5\right]}^{⊤}, \end{array}$$

for 〈*ρ*_u_〉, *σ*(*ρ*_u_), *b*, *d*, *σ*_b_ and *σ*_u_, respectively.

**Remark 3 (*θ*^old^ initialization)**

The adaptive MCMC’s *θ*^old^ is initiated with the estimates of MLE obtained using the same dataset.

**Remark 4 (convergence)**

Generally, the posterior calculated up to chain length equal to 10^6^ is virtually indistinguishable from that calculated with a chain length which is an order of magnitude longer and hence we chose to terminate the entire chain at 10^7^. In all cases this guaranteed a converged posterior. The computation time of the full chain takes 10 h or more for the identical cell case and at least 20 h for the heterogeneous cell case.

## Appendix B. Moment closures for an auto-regulatory transcriptional feedback loop in a system of identical cells

The associated (conditional) chemical master equations of the auto-regulatory transcriptional feedback loop shown in figure 1*a* are given by

$$\frac{dP_{0}(n_{p},t)}{dt}\begin{array}{rl} & =\rho_{u}\sum_{i=0}^{\infty }\frac{b^{i}}{{\left(1+b\right)}^{i+1}}P_{0}(n_{p}-i,t)-\rho_{u}P_{0}(n_{p},t)\\ & \;+d(n_{p}+1)P_{0}(n_{p}+1,t)-dn_{p}P_{0}(n_{p},t)\\ & \;-\sigma_{b}n_{p}P_{0}(n_{p},t)+\sigma_{u}P_{1}(n_{p}-1,t) \end{array}$$

$$\begin{array}{rl} \text{and}\;\frac{dP_{1}(n_{p},t)}{dt} & =d(n_{p}+1)P_{1}(n_{p}+1,t)-dn_{p}P_{1}(n_{p},t)\\ & \;+\sigma_{b}(n_{p}+1)P_{0}(n_{p}+1,t)-\sigma_{u}P_{1}(n_{p},t). \end{array}$$

The moment equations derived from these master equations are not closed and hence require a moment-closure scheme. Next, we describe the equations specifying the six types of moment-closure used in this article.

**B.1. Three moment approximation**

B 1 $$\begin{array}{rl} \partial_{t}\langle n_{p}\rangle & =(\rho_{u}b-\sigma_{u})\langle n_{g}\rangle -d\langle n_{p}\rangle -\sigma_{b}\langle n_{p}n_{g}\rangle +\sigma_{u},\\ \partial_{t}\langle n_{g}\rangle & =-\sigma_{b}\langle n_{p}n_{g}\rangle -\sigma_{u}\langle n_{g}\rangle +\sigma_{u},\\ \partial_{t}\langle n_{p}^{2}\rangle & =2\rho_{u}b\langle n_{p}n_{g}\rangle +\rho_{u}(2b^{2}+b)\langle n_{g}\rangle -2d\langle n{{\;}_{p}}^{2}\rangle +d\langle n{\;}_{p}\rangle +\sigma_{b}\langle n_{p}n_{g}\rangle -2\sigma_{b}\langle n_{p}^{2}n_{g}\rangle +\sigma_{u}(1-\langle n_{g}\rangle +2\langle n_{p}\rangle -2\langle n_{p}n_{g}\rangle ),\\ \partial_{t}\langle n_{g}^{2}\rangle & =\sigma_{b}\langle n_{p}n_{g}\rangle -2\sigma_{b}\langle n_{p}n_{g}^{2}\rangle +\sigma_{u}+\sigma_{u}\langle n_{g}\rangle -2\sigma_{u}\langle n_{g}^{2}\rangle ,\\ \partial_{t}\langle n_{p}n_{g}\rangle & =\rho_{u}b\langle n_{g}^{2}\rangle -d\langle n_{p}n_{g}\rangle +\sigma_{b}(\langle n_{p}n_{g}\rangle -\langle n_{p}n_{g}^{2}\rangle -\langle n_{p}^{2}n_{g}\rangle )+\sigma_{u}(1-\langle n_{g}^{2}\rangle +\langle n_{p}\rangle -\langle n_{p}n_{g}\rangle ),\\ \partial_{t}\langle n_{p}^{3}\rangle & =\rho_{u}b(6b^{2}+6b+1)\langle n_{g}\rangle +3\rho_{u}b(1+2b)\langle n_{p}n_{g}\rangle +3\rho_{u}b\langle n_{p}^{2}n_{g}\rangle -d\langle n_{p}\rangle +3d\langle n_{p}^{2}\rangle -3d\langle n_{p}^{3}\rangle -\sigma_{b}\langle n_{p}n_{g}\rangle +3\sigma_{b}\langle n_{p}^{2}n_{g}\rangle \\ & \;-3\sigma_{b}\langle n_{p}^{3}n_{g}\rangle +\sigma_{u}(1-\langle n_{g}\rangle +3\langle n_{p}\rangle -3\langle n_{p}n_{g}\rangle +3\langle n_{p}^{2}\rangle -3\langle n_{p}^{2}n_{g}\rangle ),\\ \partial_{t}\langle n_{p}^{2}n_{g}\rangle & =\rho_{u}b(1+2b)\langle n_{g}^{2}\rangle +2\rho_{u}b\langle n_{p}n_{g}^{2}\rangle +d\langle n_{p}n_{g}\rangle -2d\langle n_{p}^{2}n_{g}\rangle +\sigma_{b}(-\langle n_{p}n_{g}\rangle +\langle n_{p}n_{g}^{2}\rangle +2\langle n_{p}^{2}n_{g}\rangle -2\langle n_{p}^{2}n_{g}^{2}\rangle -\langle n_{p}^{3}n_{g}\rangle )\\ & \;+\sigma_{u}(1-\langle n_{g}^{2}\rangle +2\langle n_{p}\rangle -2\langle n_{p}n_{g}^{2}\rangle +\langle n_{p}^{2}\rangle -\langle n_{p}^{2}n_{g}\rangle ),\\ \partial_{t}\langle n_{p}n_{g}^{2}\rangle & =\rho_{u}b\langle n_{g}^{3}\rangle -d\langle n_{p}n_{g}^{2}\rangle +\sigma_{b}(-\langle n_{p}n_{g}\rangle +2\langle n_{p}n_{g}^{2}\rangle -\langle n_{p}n_{g}^{3}\rangle +\langle n_{p}^{2}n_{g}\rangle -2\langle n_{p}^{2}n_{g}^{2}\rangle )+\sigma_{u}(1+\langle n_{g}\rangle -\langle n_{g}^{2}\rangle -\langle n_{g}^{3}\rangle +\langle n_{p}\rangle \\ & \;+\langle n_{p}n_{g}\rangle -2\langle n_{p}n_{g}^{2}\rangle )\\ \text{and}\;\partial_{t}\langle n_{g}^{3}\rangle & =\sigma_{b}(-\langle n_{p}n_{g}\rangle +3\langle n_{p}n_{g}^{2}\rangle -3\langle n_{p}n_{g}^{3}\rangle )+\sigma_{u}(1+2\langle n_{g}\rangle -3\langle n_{g}^{3}\rangle ). \end{array}\}$$

To close the moment equations in equation (B 1) up to third order, we assume that the fourth cumulant is zero. This is enforced by the following set of algebraic equations:

$$\begin{array}{rl} \left\langle n_{p}^{3}n_{g}\right\rangle & =3\langle n_{p}n_{g}\rangle (-2{\left\langle n_{p}\right\rangle }^{2}+\langle n_{p}^{2}\rangle )+\langle n_{g}\rangle (\langle n_{p}^{3}\rangle +6{\left\langle n_{p}\right\rangle }^{3}-6\langle n_{p}\rangle \;\langle n_{p}^{2}\rangle )+3\langle n_{p}\rangle \;\langle n_{p}^{2}n_{g}\rangle ,\\ \left\langle n_{p}n_{g}^{3}\right\rangle & =6{\left\langle n_{g}\right\rangle }^{3}\langle n_{p}\rangle -6\langle n_{g}^{2}\rangle \;\langle n_{p}n_{g}\rangle +3\langle n_{p}n_{g}\rangle \;\langle n_{g}^{2}\rangle +3\langle n_{g}\rangle (-2\langle n_{p}\rangle \;\langle n_{g}^{2}\rangle +\langle n_{p}n_{g}^{2}\rangle )+\langle n_{p}\rangle \;\langle n_{g}^{3}\rangle \\ \text{and}\;\langle n_{p}^{2}n_{g}^{2}\rangle & =2({\left\langle n_{p}n_{g}\right\rangle }^{2}-{\left\langle n_{p}\right\rangle }^{2}\langle n_{g}^{2}\rangle +\langle n_{p}\rangle \;\langle n_{p}n_{g}^{2}\rangle +\langle n_{g}\rangle (-4\langle n_{p}\rangle \;\langle n_{p}n_{g}\rangle +\langle n_{p}^{2}n_{g}\rangle ))+{\left\langle n_{g}\right\rangle }^{2}(6{\left\langle n_{p}\right\rangle }^{2}-2\langle n_{p}^{2}\rangle )+\langle n_{p}^{2}\rangle \;\langle n_{g}^{2}\rangle . \end{array}\}$$

**B.2. Linear-mapping approximation**

To use the LMA to close the moment equations, we derive the moment equations for the equivalent linear gene regulatory network (this is done by replacing the bimolecular reaction $G+P\overset{\sigma_{b}}{⟶}G^{*}$ by the first-order reaction $G\overset{{\bar{\sigma }}_{b}}{⟶}G^{*}$):

$$\begin{array}{rl} \partial_{t}\langle n_{p}\rangle & =\rho_{u}b\langle n_{g}\rangle -d\langle n_{p}\rangle ,\\ \partial_{t}\langle n_{g}\rangle & =-{\bar{\sigma }}_{b}\langle n_{g}\rangle +\sigma_{u}(1-\langle n_{g}\rangle ),\\ \partial_{t}\langle n_{p}n_{g}\rangle & =\rho_{u}b\langle n_{g}\rangle +\sigma_{u}\langle n_{p}\rangle -(d+{\bar{\sigma }}_{b}+\sigma_{u})\langle n_{p}n_{g}\rangle ,\\ \partial_{t}\langle n_{p}^{2}\rangle & =2\rho_{u}b\langle n_{p}n_{g}\rangle +\rho_{u}b(1+2b)\langle n_{g}\rangle -2d\langle n_{p}^{2}\rangle +d\langle n_{p}\rangle ,\\ \partial_{t}\langle n_{p}^{2}n_{g}\rangle & =(2\rho_{u}b+d)\langle n_{p}n_{g}\rangle +\rho_{u}b(1+2b)\langle n_{g}\rangle -(2d+\sigma_{u}+{\bar{\sigma }}_{b})\langle n_{p}^{2}n_{g}\rangle +\sigma_{u}\langle n_{p}^{2}\rangle \\ \text{and}\;\;\;\partial_{t}\langle n_{p}^{3}\rangle & =3\rho_{u}b\langle n_{p}^{2}n_{g}\rangle +3\rho_{u}b(1+2b)\langle n_{p}n_{g}\rangle +\rho_{u}b(1+6b+6b^{2})\langle n_{g}\rangle -d\langle n_{p}\rangle +3d\langle n_{p}^{2}\rangle -3d\langle n_{p}^{3}\rangle , \end{array}\}$$

where the equivalent parameter ${\bar{\sigma }}_{b}$ is determined by

$${\bar{\sigma }}_{b}=\sigma_{b}\frac{\left\langle n_{p}n_{g}\right\rangle }{\left\langle n_{g}\right\rangle }.$$

**B.3. Conditional derivative matching**

The moment equations are

B 2 $$\begin{array}{rl} \partial_{t}\langle n_{p}\rangle & =(\rho_{u}b-\sigma_{u})\langle n_{g}\rangle -d\langle n_{p}\rangle -\sigma_{b}\langle n_{p}n_{g}\rangle +\sigma_{u},\\ \partial_{t}\langle n_{g}\rangle & =-\sigma_{b}\langle n_{p}n_{g}\rangle -\sigma_{u}\langle n_{g}\rangle +\sigma_{u},\\ \partial_{t}\langle n_{p}^{2}\rangle & =2\rho_{u}b\langle n_{p}n_{g}\rangle +\rho_{u}(2b^{2}+b)\langle n_{g}\rangle -2d\langle n_{p}^{2}\rangle +d\langle n_{p}\rangle +\sigma_{b}\langle n_{p}n_{g}\rangle -2\sigma_{b}\langle n_{p}^{2}n_{g}\rangle +\sigma_{u}(1-\langle n_{g}\rangle +2\langle n_{p}\rangle -2\langle n_{p}n_{g}\rangle ),\\ \partial_{t}\langle n_{p}n_{g}\rangle & =\rho_{u}b\langle n_{g}\rangle -d\langle n_{p}n_{g}\rangle -\sigma_{b}\langle n_{p}^{2}n_{g}\rangle +\sigma_{u}(1-\langle n_{g}\rangle +\langle n_{p}\rangle -\langle n_{p}n_{g}\rangle ),\\ \partial_{t}\langle n_{p}^{3}\rangle & =\rho_{u}b(6b^{2}+6b+1)\langle n_{g}\rangle +3\rho_{u}b(1+2b)\langle n_{p}n_{g}\rangle +3\rho_{u}b\langle n_{p}^{2}n_{g}\rangle -d\langle n_{p}\rangle +3d\langle n_{p}^{2}\rangle -3d\langle n_{p}^{3}\rangle -\sigma_{b}\langle n_{p}n_{g}\rangle +3\sigma_{b}\langle n_{p}^{2}n_{g}\rangle \\ & \;-3\sigma_{b}\langle n_{p}^{3}n_{g}\rangle +\sigma_{u}(1-\langle n_{g}\rangle +3\langle n_{p}\rangle -3\langle n_{p}n_{g}\rangle +3\langle n_{p}^{2}\rangle -3\langle n_{p}^{2}n_{g}\rangle )\\ \text{and}\;\partial_{t}\langle n_{p}^{2}n_{g}\rangle & =\rho_{u}b(1+2b)\langle n_{g}\rangle +(2\rho_{u}b+d)\langle n_{p}n_{g}\rangle -2d\langle n_{p}^{2}n_{g}\rangle -\sigma_{b}\langle n_{p}^{3}n_{g}\rangle +\sigma_{u}(1-\langle n_{g}\rangle +2\langle n_{p}\rangle -2\langle n_{p}n_{g}\rangle +\langle n_{p}^{2}\rangle -\langle n_{p}^{2}n_{g}\rangle ), \end{array}\}$$

and they are closed by approximating $\left\langle n_{p}^{3}n_{g}\right\rangle$ by

$$\langle n_{p}^{3}n_{g}\rangle ={\left(\frac{\left\langle n_{p}^{2}n_{g}\right\rangle }{\left\langle n_{p}n_{g}\right\rangle }\right)}^{3}\langle n_{g}\rangle .$$

**B.4. Derivative matching**

DM proposes to close (B 2) by the means of

$$\langle n_{p}^{3}n_{g}\rangle ={\left(\frac{\left\langle n_{p}^{2}n_{g}\right\rangle }{\left\langle n_{p}n_{g}\right\rangle }\right)}^{3}{\left(\frac{\left\langle n_{p}\right\rangle }{\left\langle n_{p}^{2}\right\rangle }\right)}^{3}\langle n_{p}^{3}\rangle \;\langle n_{g}\rangle .$$

**B.5. Conditional Gaussian**

CG proposes to close (B 2) by the means of

$$\langle n_{p}^{3}n_{g}\rangle =\frac{3\langle n_{p}^{2}n_{g}\rangle \;\langle n_{p}n_{g}\rangle }{\left\langle n_{g}\right\rangle }-\frac{2{\left\langle n_{p}n_{g}\right\rangle }^{3}}{{\left\langle n_{g}\right\rangle }^{2}}.$$

**B.6. Linear-noise approximation**

The mean numbers of protein and gene, 〈*n*_p_〉 and 〈*n*_g_〉, respectively, follow the deterministic rate equations:

B 3 $$\begin{array}{rl} \partial_{t}\langle n_{p}\rangle & =\rho_{u}b\langle n_{g}\rangle -d\langle n_{p}\rangle -\sigma_{b}\langle n_{p}\rangle \;\langle n_{g}\rangle +\sigma_{u}(1-\langle n_{g}\rangle )\;\text{and}\\ \partial_{t}\langle n_{g}\rangle & =-\sigma_{b}\langle n_{p}\rangle \;\langle n_{g}\rangle +\sigma_{u}(1-\langle n_{g}\rangle ). \end{array}$$

The second-order central moments follow the following Lyapunov equation:

$$\partial_{t}\Sigma =J\Sigma +\Sigma J^{⊤}+D,$$

where

$$\begin{array}{rl} J & =\left[\begin{array}{cc} -d-\sigma_{b}\langle n_{g}\rangle & \rho_{u}b-\sigma_{b}\langle n_{p}\rangle -\sigma_{u}\\ -\sigma_{b}\langle n_{g}\rangle & -\sigma_{b}\langle n_{p}\rangle -\sigma_{u} \end{array}\right],\\ D & =\left[\begin{array}{cc} \rho_{u}b(1+2b)\langle n_{g}\rangle +d\langle n_{p}\rangle +\sigma_{b}\langle n_{p}\rangle \;\langle n_{g}\rangle +\sigma_{u}(1-\langle n_{g}\rangle ) & \sigma_{b}\langle n_{p}\rangle \;\langle n_{g}\rangle +\sigma_{u}(1-\langle n_{g}\rangle )\\ \sigma_{b}\langle n_{p}\rangle \;\langle n_{g}\rangle +\sigma_{u}(1-\langle n_{g}\rangle ) & \sigma_{b}\langle n_{p}\rangle \;\langle n_{g}\rangle +\sigma_{u}(1-\langle n_{g}\rangle ) \end{array}\right] \end{array}$$

and

$$\Sigma =\left[\begin{array}{cc} \mathrm{cov}(n_{p},n_{p}) & \mathrm{cov}(n_{p},n_{g})\\ \mathrm{cov}(n_{p},n_{g}) & \mathrm{cov}(n_{g},n_{g}) \end{array}\right].$$

## Appendix C. Moment closures for an auto-regulatory transcriptional feedback loop in a system of non-identical cells

**C.1. Conditional derivative matching**

The moment equation are

$$\begin{array}{rl} \partial_{t}\langle n_{p}\rangle & =b\langle \rho_{u}n_{g}\rangle -d\langle n_{p}\rangle -\sigma_{b}\langle n_{p}n_{g}\rangle -\sigma_{u}\langle n_{g}\rangle +\sigma_{u},\\ \partial_{t}\langle n_{g}\rangle & =-\sigma_{b}\langle n_{p}n_{g}\rangle -\sigma_{u}\langle n_{g}\rangle +\sigma_{u},\\ \partial_{t}\langle n_{p}^{2}\rangle & =2b\langle \rho_{u}n_{p}n_{g}\rangle +(2b^{2}+b)\langle \rho_{u}n_{g}\rangle -2d\langle n_{p}^{2}\rangle +d\langle n_{p}\rangle +\sigma_{b}\langle n_{p}n_{g}\rangle -2\sigma_{b}\langle n_{p}^{2}n_{g}\rangle +\sigma_{u}(1-\langle n_{g}\rangle +2\langle n_{p}\rangle -2\langle n_{p}n_{g}\rangle ),\\ \partial_{t}\langle n_{p}n_{g}\rangle & =b\langle \rho_{u}n_{g}\rangle -d\langle n_{p}n_{g}\rangle -\sigma_{b}\langle n_{p}^{2}n_{g}\rangle +\sigma_{u}(1-\langle n_{g}\rangle +\langle n_{p}\rangle -\langle n_{p}n_{g}\rangle ),\\ \partial_{t}\langle n_{p}^{2}n_{g}\rangle & =b(1+2b)\langle \rho_{u}n_{g}\rangle +2b\langle \rho_{u}n_{p}n_{g}\rangle +d\langle n_{p}n_{g}\rangle -2d\langle n_{p}^{2}n_{g}\rangle -\sigma_{b}\langle n_{p}^{3}n_{g}\rangle +\sigma_{u}(1-\langle n_{g}\rangle +2\langle n_{p}\rangle -2\langle n_{p}n_{g}\rangle +\langle n_{p}^{2}\rangle -\langle n_{p}^{2}n_{g}\rangle ),\\ \partial_{t}\langle \rho_{u}n_{p}\rangle & =b\langle \rho_{u}^{2}n_{g}\rangle -d\langle \rho_{u}n_{p}\rangle -\sigma_{b}\langle \rho_{u}n_{p}n_{g}\rangle -\sigma_{u}\langle \rho_{u}n_{g}\rangle +\sigma_{u}\langle \rho_{u}\rangle ,\\ \partial_{t}\langle \rho_{u}n_{g}\rangle & =-\sigma_{b}\langle \rho_{u}n_{p}n_{g}\rangle -\sigma_{u}\langle \rho_{u}n_{g}\rangle +\sigma_{u}\langle \rho_{u}\rangle ,\\ \partial_{t}\langle \rho_{u}n_{p}n_{g}\rangle & =b\langle \rho_{u}^{2}n_{g}\rangle -d\langle \rho_{u}n_{p}n_{g}\rangle -\sigma_{b}\langle \rho_{u}n_{p}^{2}n_{g}\rangle +\sigma_{u}(\langle \rho_{u}\rangle -\langle \rho_{u}n_{g}\rangle +\langle \rho_{u}n_{p}\rangle -\langle \rho_{u}n_{p}n_{g}\rangle )\\ \text{and}\;\partial_{t}\langle \rho_{u}^{2}n_{g}\rangle & =-\sigma_{b}\langle \rho_{u}^{2}n_{p}n_{g}\rangle -\sigma_{u}\langle \rho_{u}^{2}n_{g}\rangle +\sigma_{u}\langle \rho_{u}^{2}\rangle , \end{array}\}$$

which are closed by the following algebraic equations:

$$\begin{array}{rl} \left\langle \rho_{u}n_{p}^{2}n_{g}\right\rangle & =\left(\frac{\left\langle n_{p}^{2}n_{g}\right\rangle }{\left\langle \rho_{u}n_{g}\right\rangle }\right){\left(\frac{\left\langle \rho_{u}n_{p}n_{g}\right\rangle }{\left\langle n_{p}n_{g}\right\rangle }\right)}^{2}\langle n_{g}\rangle ,\\ \left\langle \rho_{u}^{2}n_{p}n_{g}\right\rangle & =\left(\frac{\left\langle \rho_{u}^{2}n_{g}\right\rangle }{\left\langle n_{p}n_{g}\right\rangle }\right){\left(\frac{\left\langle \rho_{u}n_{p}n_{g}\right\rangle }{\left\langle \rho_{u}n_{g}\right\rangle }\right)}^{2}\langle n_{g}\rangle \\ \text{and}\;\;\langle n_{p}^{2}n_{g}\rangle & ={\left(\frac{\left\langle n_{p}^{2}n_{g}\right\rangle }{\left\langle n_{p}n_{g}\right\rangle }\right)}^{3}\langle n_{g}\rangle . \end{array}\}$$

**C.2. Linear-mapping approximation**

The moment equations are

$$\begin{array}{rl} & \partial_{t}\langle n_{p}\rangle =b\langle \rho_{u}n_{g}\rangle -d\langle n_{p}\rangle ,\\ & \partial_{t}\langle n_{g}\rangle =-{\bar{\sigma }}_{b}\langle n_{g}\rangle +\sigma_{u}(1-\langle n_{g}\rangle ),\\ & \partial_{t}\langle n_{p}^{2}\rangle =2b\langle \rho_{u}n_{p}n_{g}\rangle +b(1+2b)\langle \rho_{u}n_{g}\rangle -2d\langle n_{p}^{2}\rangle +d\langle n_{p}\rangle ,\\ & \partial_{t}\langle n_{p}n_{g}\rangle =b\langle \rho_{u}n_{g}\rangle +\sigma_{u}\langle n_{p}\rangle -(d+{\bar{\sigma }}_{b}+\sigma_{u})\langle n_{p}n_{g}\rangle ,\\ & \partial_{t}\langle \rho_{u}n_{p}\rangle =b\langle \rho_{u}^{2}n_{g}\rangle -d\langle \rho_{u}n_{p}\rangle ,\\ & \partial_{t}\langle \rho_{u}n_{g}\rangle =-{\bar{\sigma }}_{b}\langle \rho_{u}n_{g}\rangle +\sigma_{u}(\langle \rho_{u}\rangle -\langle \rho_{u}n_{g}\rangle ),\\ & \partial_{t}\langle \rho_{u}^{2}n_{g}\rangle =-{\bar{\sigma }}_{b}\langle \rho_{u}^{2}n_{g}\rangle +\sigma_{u}(\langle \rho_{u}^{2}\rangle -\langle \rho_{u}^{2}n_{g}\rangle )\\ \text{and}\; & \partial_{t}\langle \rho_{u}n_{p}n_{g}\rangle =b\langle \rho_{u}^{2}n_{g}\rangle +\sigma_{u}\langle \rho_{u}n_{p}\rangle -(d+{\bar{\sigma }}_{b}+\sigma_{u})\langle \rho_{u}n_{p}n_{g}\rangle , \end{array}\}$$

in which ${\bar{\sigma }}_{b}$ is determined by

$${\bar{\sigma }}_{b}=\sigma_{b}\frac{\left\langle n_{p}n_{g}\right\rangle }{\left\langle n_{g}\right\rangle }.$$

**C.3. Three moment approximation**

The moment equations used are

$$\begin{array}{rl} \partial_{t}\langle n_{p}\rangle & =b\langle \rho_{u}n_{g}\rangle -d\langle n_{p}\rangle -\sigma_{b}\langle n_{p}n_{g}\rangle -\sigma_{u}\langle n_{g}\rangle +\sigma_{u},\\ \partial_{t}\langle n_{g}\rangle & =-\sigma_{b}\langle n_{p}n_{g}\rangle -\sigma_{u}\langle n_{g}\rangle +\sigma_{u},\\ \partial_{t}\langle n_{p}^{2}\rangle & =2b\langle \rho_{u}n_{p}n_{g}\rangle +(2b^{2}+b)\langle \rho_{u}n_{g}\rangle -2d\langle n_{p}^{2}\rangle +d\langle n_{p}\rangle +\sigma_{b}\langle n_{p}n_{g}\rangle -2\sigma_{b}\langle n_{p}^{2}n_{g}\rangle +\sigma_{u}(1-\langle n_{g}\rangle +2\langle n_{p}\rangle -2\langle n_{p}n_{g}\rangle ),\\ \partial_{t}\langle n_{p}n_{g}\rangle & =b\langle \rho_{u}n_{g}^{2}\rangle -d\langle n_{p}n_{g}\rangle +\sigma_{b}(\langle n_{p}n_{g}\rangle -\langle n_{p}n_{g}^{2}\rangle -\langle n_{p}^{2}n_{g}\rangle )+\sigma_{u}(1-\langle n_{g}^{2}\rangle +\langle n_{p}\rangle -\langle n_{p}n_{g}\rangle ),\\ \partial_{t}\langle n_{g}^{2}\rangle & =\sigma_{b}\langle n_{p}n_{g}\rangle -2\sigma_{b}\langle n_{p}n_{g}^{2}\rangle +\sigma_{u}+\sigma_{u}\langle n_{g}\rangle -2\sigma_{u}\langle n_{g}^{2}\rangle ,\\ \partial_{t}\langle n_{p}^{3}\rangle & =b(6b^{2}+6b+1)\langle \rho_{u}n_{g}\rangle +3b(1+2b)\langle \rho_{u}n_{p}n_{g}\rangle +3b\langle \rho_{u}n_{p}^{2}n_{g}\rangle -d\langle n_{p}\rangle +3d\langle n_{p}^{2}\rangle -3d\langle n_{p}^{3}\rangle \\ & \;-\sigma_{b}\langle n_{p}n_{g}\rangle +3\sigma_{b}\langle n_{p}^{2}n_{g}\rangle -3\sigma_{b}\langle n_{p}^{3}n_{g}\rangle +\sigma_{u}(1-\langle n_{g}\rangle +3\langle n_{p}\rangle -3\langle n_{p}n_{g}\rangle +3\langle n_{p}^{2}\rangle -3\langle n_{p}^{2}n_{g}\rangle ),\\ \partial_{t}\langle n_{p}^{2}ng\rangle & =b(1+2b)\langle \rho_{u}n_{g}^{2}\rangle +2b\langle \rho_{u}n_{p}n_{g}^{2}\rangle +d\langle n_{p}n_{g}\rangle -2d\langle n_{p}^{2}n_{g}\rangle \\ & \;+\sigma_{b}(-\langle n_{p}n_{g}\rangle +\langle n_{p}n_{g}^{2}\rangle +2\langle n_{p}^{2}n_{g}\rangle -2\langle n_{p}^{2}n_{g}^{2}\rangle -\langle n_{p}^{3}n_{g}\rangle )\\ & \;+\sigma_{u}(1-\langle n_{g}^{2}\rangle +2\langle n_{p}\rangle -2\langle n_{p}n_{g}^{2}\rangle +\langle n_{p}^{2}\rangle -\langle n_{p}^{2}n_{g}\rangle ),\\ \partial_{t}\langle n_{p}n_{g}^{2}\rangle & =b\langle \rho_{u}n_{g}^{3}\rangle -d\langle n_{p}n_{g}^{2}\rangle +\sigma_{b}(-\langle n_{p}n_{g}\rangle +2\langle n_{p}n_{g}^{2}\rangle -\langle n_{p}n_{g}^{3}\rangle +\langle n_{p}^{2}n_{g}\rangle -2\langle n_{p}^{2}n_{g}^{2}\rangle )\\ & \;+\sigma_{u}(1+\langle n_{g}\rangle -\langle n_{g}^{2}\rangle -\langle n_{g}^{3}\rangle +\langle n_{p}\rangle +\langle n_{p}n_{g}\rangle -2\langle n_{p}n_{g}^{2}\rangle ),\\ \partial_{t}\langle n_{g}^{3}\rangle & =\sigma_{b}(-\langle n_{p}n_{g}\rangle +3\langle n_{p}n_{g}^{2}\rangle -3\langle n_{p}n_{g}^{3}\rangle )+\sigma_{u}(1+2\langle n_{g}\rangle -3\langle n_{g}^{3}\rangle ),\\ \partial_{t}\langle \rho_{u}n_{p}\rangle & =b\langle \rho_{u}^{2}n_{g}\rangle -d\langle \rho_{u}n_{p}\rangle -\sigma_{b}\langle \rho_{u}n_{p}n_{g}\rangle -\sigma_{u}\langle \rho_{u}n_{g}\rangle +\sigma_{u}\langle \rho_{u}\rangle ,\\ \partial_{t}\langle \rho_{u}n_{g}\rangle & =-\sigma_{b}\langle \rho_{u}n_{p}n_{g}\rangle -\sigma_{u}\langle \rho_{u}n_{g}\rangle +\sigma_{u}\langle \rho_{u}\rangle ,\\ \partial_{t}\langle \rho_{u}^{2}n_{p}\rangle & =b\langle \rho_{u}^{3}n_{g}\rangle -d\langle \rho_{u}^{2}n_{p}\rangle -\sigma_{b}\langle \rho_{u}^{2}n_{p}n_{g}\rangle -\sigma_{u}\langle \rho_{u}^{2}n_{g}\rangle +\sigma_{u}\langle \rho_{u}^{2}\rangle ,\\ \partial_{t}\langle \rho_{u}^{2}n_{g}\rangle & =-\sigma_{b}\langle \rho_{u}^{2}n_{p}n_{g}\rangle -\sigma_{u}\langle \rho_{u}^{2}n_{g}\rangle +\sigma_{u}\langle \rho_{u}^{2}\rangle ,\\ \partial_{t}\langle \rho_{u}n_{p}^{2}\rangle & =2b\langle \rho_{u}^{2}n_{p}n_{g}\rangle +(2b^{2}+b)\langle \rho_{u}^{2}n_{g}\rangle -2d\langle \rho_{u}n_{p}^{2}\rangle +d\langle \rho_{u}n_{p}\rangle +\sigma_{b}\langle \rho_{u}n_{p}n_{g}\rangle -2\sigma_{b}\langle \rho_{u}n_{p}^{2}n_{g}\rangle +\sigma_{u}(\langle \rho_{u}\rangle -\langle \rho_{u}n_{g}\rangle +2\langle \rho_{u}n_{p}\rangle \\ & \;-2\langle \rho_{u}n_{p}n_{g}\rangle ),\\ \partial_{t}\langle \rho_{u}n_{p}n_{g}\rangle & =b\langle \rho_{u}^{2}n_{g}^{2}\rangle -d\langle \rho_{u}n_{p}n_{g}\rangle +\sigma_{b}(\langle \rho_{u}n_{p}n_{g}\rangle -\langle \rho_{u}n_{p}n_{g}^{2}\rangle -\langle \rho_{u}n_{p}^{2}n_{g}\rangle )+\sigma_{u}(\langle \rho_{u}\rangle -\langle \rho_{u}n_{g}^{2}\rangle +\langle \rho_{u}n_{p}\rangle -\langle \rho_{u}n_{p}n_{g}\rangle )\\ \text{and}\;\partial_{t}\langle \rho_{u}n_{g}^{2}\rangle & =\sigma_{b}\langle \rho_{u}n_{p}n_{g}\rangle -2\sigma_{b}\langle \rho_{u}n_{p}n_{g}^{2}\rangle +\sigma_{u}\langle \rho_{u}\rangle +\sigma_{u}\langle \rho_{u}n_{g}\rangle -2\sigma_{u}\langle \rho_{u}n_{g}^{2}\rangle , \end{array}\}$$

which are closed by

$$\begin{array}{rl} \left\langle n_{p}^{3}n_{g}\right\rangle & =3\langle n_{p}n_{g}\rangle (-2{\left\langle n_{p}\right\rangle }^{2}+\langle n_{p}^{2}\rangle )+\langle n_{g}\rangle (\langle n_{p}^{3}\rangle +6{\left\langle n_{p}\right\rangle }^{3}-6\langle n_{p}\rangle \;\langle n_{p}^{2}\rangle )+3\langle n_{p}\rangle \;\langle n_{p}^{2}n_{g}\rangle ,\\ \left\langle n_{p}n_{g}^{3}\right\rangle & =6{\left\langle n_{g}\right\rangle }^{3}\langle n_{p}\rangle -6\langle n_{g}^{2}\rangle \;\langle n_{p}n_{g}\rangle +3\langle n_{p}n_{g}\rangle \;\langle n_{g}^{2}\rangle +3\langle n_{g}\rangle (-2\langle n_{p}\rangle \;\langle n_{g}^{2}\rangle +\langle n_{p}n_{g}^{2}\rangle )+\langle n_{p}\rangle \;\langle n_{g}^{3}\rangle ,\\ \left\langle n_{p}^{2}n_{g}^{2}\right\rangle & =2({\left\langle n_{p}n_{g}\right\rangle }^{2}-{\left\langle n_{p}\right\rangle }^{2}\langle n_{g}^{2}\rangle +\langle n_{p}\rangle \;\langle n_{p}n_{g}^{2}\rangle +\langle n_{g}\rangle (-4\langle n_{p}\rangle \;\langle n_{p}n_{g}\rangle +\langle n_{p}^{2}n_{g}\rangle ))+{\left\langle n_{g}\right\rangle }^{2}(6{\left\langle n_{p}\right\rangle }^{2}-2\langle n_{p}^{2}\rangle )+\langle n_{p}^{2}\rangle \;\langle n_{g}^{2}\rangle ,\\ \left\langle \rho_{u}n_{p}^{2}n_{g}\right\rangle & =2\langle \rho_{u}n_{p}n_{g}\rangle \;\langle n_{p}\rangle +\langle \rho_{u}n_{g}\rangle \;\langle n_{p}^{2}\rangle +\langle \rho_{u}\rangle \;\langle n_{p}^{2}n_{g}\rangle +2\langle \rho_{u}n_{p}\rangle \;\langle n_{p}n_{g}\rangle +\langle \rho_{u}n_{p}^{2}\rangle \;\langle n_{g}\rangle \\ & \;-2(\langle \rho_{u}n_{g}\rangle \;{\left\langle n_{p}\right\rangle }^{2}+2\langle n_{p}n_{g}\rangle \;\langle \rho_{u}\rangle \;\langle n_{p}\rangle +2\langle \rho_{u}n_{p}\rangle \;\langle n_{p}\rangle \;\langle n_{g}\rangle +\langle n_{g}\rangle \;\langle \rho_{u}\rangle \;\langle n_{p}^{2}\rangle -3\langle n_{g}\rangle \;\langle \rho_{u}\rangle \;{\langle n_{p}\rangle }^{2}),\\ \left\langle \rho_{u}n_{p}n_{g}^{2}\right\rangle & =2\langle \rho_{u}n_{p}n_{g}\rangle \;\langle n_{g}\rangle +\langle \rho_{u}n_{p}\rangle \;\langle n_{g}^{2}\rangle +\langle \rho_{u}\rangle \;\langle n_{p}n_{g}^{2}\rangle +2\langle \rho_{u}n_{g}\rangle \;\langle n_{p}n_{g}\rangle +\langle \rho_{u}n_{g}^{2}\rangle \;\langle n_{p}\rangle \\ & \;-2(\langle \rho_{u}n_{p}\rangle \;{\langle n_{g}\rangle }^{2}+2\langle n_{p}n_{g}\rangle \;\langle \rho_{u}\rangle \;\langle n_{g}\rangle +2\langle \rho_{u}n_{g}\rangle \;\langle n_{p}\rangle \;\langle n_{g}\rangle +\langle n_{p}\rangle \;\langle \rho_{u}\rangle \;\langle n_{g}^{2}\rangle -3\langle n_{p}\rangle \;\langle \rho_{u}\rangle \;{\langle n_{g}\rangle }^{2}),\\ \left\langle \rho_{u}^{2}n_{p}n_{g}\right\rangle & =2\langle \rho_{u}n_{p}n_{g}\rangle \;\langle \rho_{u}\rangle +\langle n_{p}n_{g}\rangle \;\langle \rho_{u}^{2}\rangle +\langle n_{g}\rangle \;\langle \rho_{u}^{2}n_{p}\rangle +2\langle \rho_{u}n_{g}\rangle \;\langle \rho_{u}n_{p}\rangle +\langle \rho_{u}^{2}n_{g}\rangle \;\langle n_{p}\rangle \\ & \;-2(\langle n_{p}n_{g}\rangle \;{\left\langle \rho_{u}\right\rangle }^{2}+2\langle \rho_{u}n_{p}\rangle \;\langle \rho_{u}\rangle \;\langle n_{g}\rangle +2\langle \rho_{u}n_{g}\rangle \;\langle n_{p}\rangle \;\langle \rho_{u}\rangle +\langle n_{p}\rangle \;\langle n_{g}\rangle \;\langle \rho_{u}^{2}\rangle -3\langle n_{p}\rangle \;\langle n_{g}\rangle \;{\left\langle \rho_{u}\right\rangle }^{2}),\\ \left\langle \rho_{u}n_{g}^{3}\right\rangle & =6{\left\langle n_{g}\right\rangle }^{3}\langle \rho_{u}\rangle -6\langle n_{g}^{2}\rangle \;\langle \rho_{u}n_{g}\rangle +3\langle \rho_{u}n_{g}\rangle \;\langle n_{g}^{2}\rangle +3\langle n_{g}\rangle (-2\langle \rho_{u}\rangle \;\langle n_{g}^{2}\rangle +\langle \rho_{u}n_{g}^{2}\rangle )+\langle \rho_{u}\rangle \;\langle n_{g}^{3}\rangle ,\\ \left\langle \rho_{u}^{2}n_{g}^{2}\right\rangle & =2({\left\langle \rho_{u}n_{g}\right\rangle }^{2}-2{\left\langle \rho_{u}\right\rangle }^{2}\langle n_{g}^{2}\rangle +\langle \rho_{u}\rangle \;\langle \rho_{u}n_{g}^{2}\rangle +\langle n_{g}\rangle (-4\langle \rho_{u}\rangle \;\langle \rho_{u}n_{g}\rangle +\langle \rho_{u}^{2}n_{g}\rangle ))+{\left\langle n_{g}\right\rangle }^{2}(6{\left\langle \rho_{u}\right\rangle }^{2}-2\langle \rho_{u}^{2}\rangle )+\langle \rho_{u}^{2}\rangle \;\langle n_{g}^{2}\rangle \\ \text{and}\;\langle \rho_{u}^{3}n_{g}\rangle & =3\langle \rho_{u}n_{g}\rangle (-2{\left\langle \rho_{u}\right\rangle }^{2}+\langle \rho_{u}^{2}\rangle )+\langle n_{g}\rangle (\langle \rho_{u}^{3}\rangle +6{\left\langle \rho_{u}\right\rangle }^{3}-6\langle \rho_{u}\rangle \;\langle \rho_{u}^{2}\rangle )+3\langle \rho_{u}\rangle \;\langle \rho_{u}^{2}n_{g}\rangle . \end{array}\}$$
