# Supplementary material for: Accuracy of parameter estimation for auto-regulatory transcriptional feedback loops from noisy data
Source: J R Soc Interface. 2019 Apr 3;16(153):20180967. doi: 10.1098/rsif.2018.0967 (PMC6505555; doi:10.1098/rsif.2018.0967)
Supplement: Supplementary Information [file rsif20180967supp1.pdf]

Supplemental Figures:
Accuracy of parameter estimation for auto-regulatory transcriptional feedback loops from noisy data

Zhixing Cao, Ramon Grima

School of Biological Sciences, the University of Edinburgh, Max Born Crescent Edinburgh, EH9 3BF, Scotland, United Kingdom

March 5, 2019

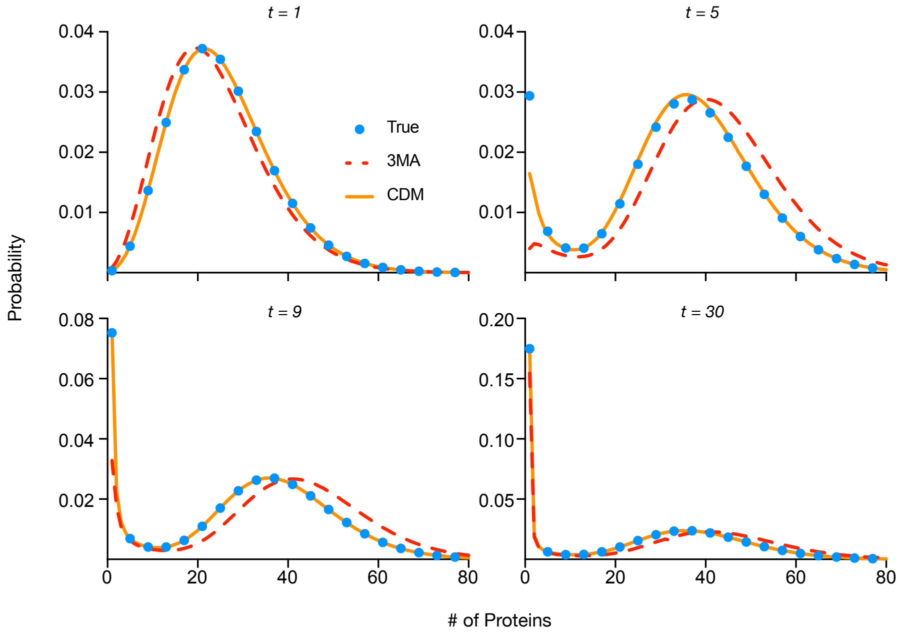

Figure 1: Distribution reconstruction based on inferred kinetic parameters. The distributions at time $t = 1, 5, 9, 30$ are reconstructed using the FSP algorithm fed with MLE parameter estimates from two different moment closure techniques (3MA and CDM). These are contrasted with the distribution computed using the true parameters. The parameter estimates for the two closures can be found in Table 1 in the main text.

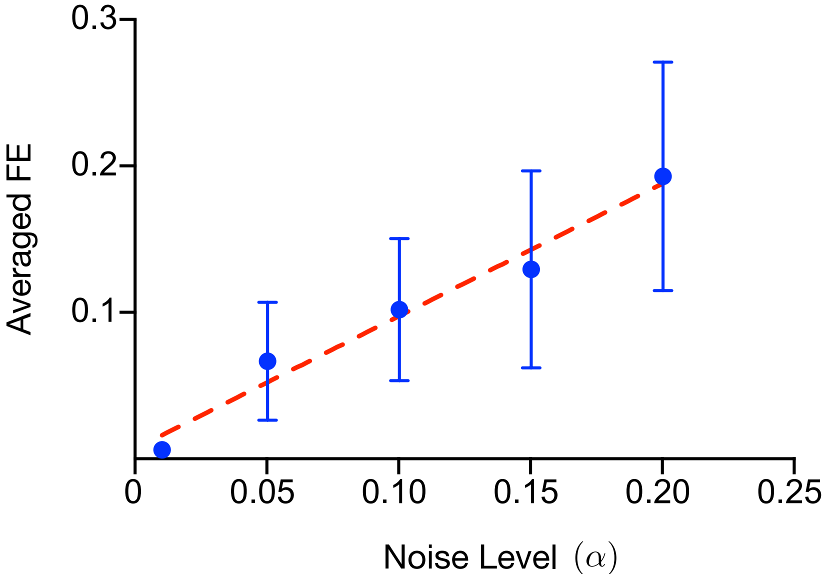

Figure 2: Robustness of inference against external noise. The moment data from the SSA is multiplied by a factor $1 + \epsilon$ where ϵ is a uniform random number between $-\alpha$ and α. The MLE (using the LMA closure) is used to infer parameters (as in Table 1 in the main text) and the fractional error averaged over all parameters is computed as a function of the noise level, α. For each noise level, ten independent replicate inference experiments are performed yielding a set of ten average fractional errors; the average and standard deviation of this set is plotted as a function of α in the plot.

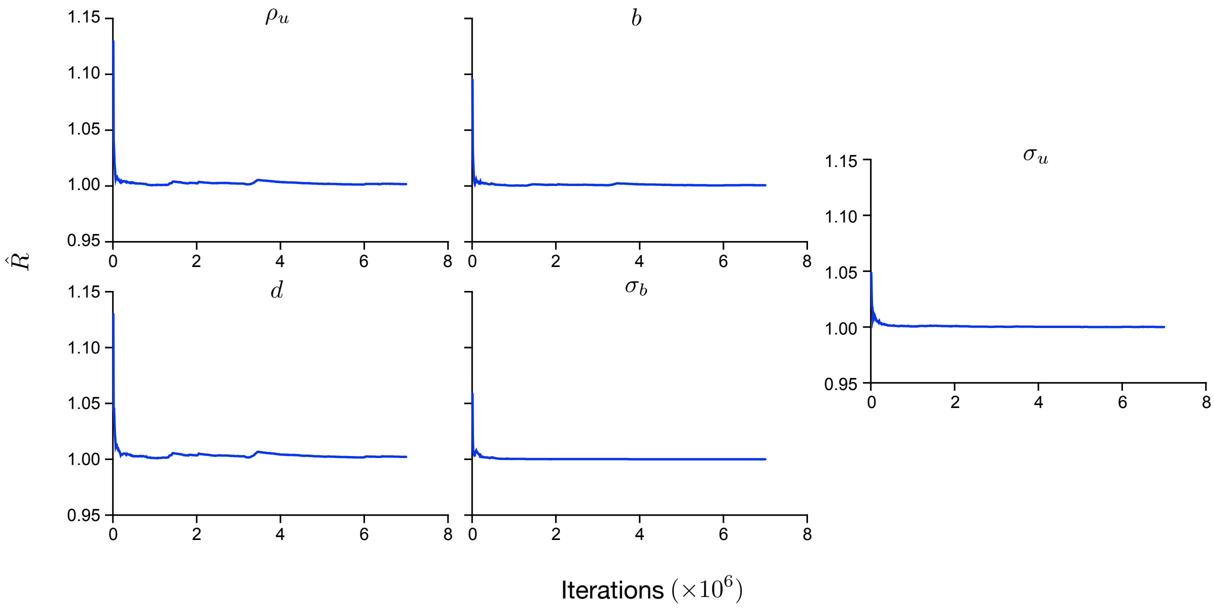

Figure 3: Convergence of the MCMC chain. We plot the Gelman-Rubin ratio ($\hat{R}$) as a function of the number of iterations for an MCMC chain using moment equations closed by the LMA. The parameters are as in Fig. 4 in the main text. Here we randomly select 10 overdispersed starting points from the prior distribution and calculate $\hat{R}$ every 10^4 iterations after the initial burnin period (3×10^6). The indices for all parameters quickly tend to 1 in 2×10^6 iterations thus clearly showing rapid chain convergence.
